# Determining Factors to Understand the External Quantum Efficiency Values: Study Carried Out with Copper(I)-I and 1,2-Bis(4-pyridyl)ethane Coordination Polymers as Downshifters in Photovoltaic Modules

**DOI:** 10.1021/acs.inorgchem.3c04232

**Published:** 2024-03-01

**Authors:** Andrea García-Hernán, Gabriela Brito-Santos, Elena de la Rubia, Fernando Aguilar-Galindo, Oscar Castillo, Ginés Lifante-Pedrola, Joaquín Sanchiz, Ricardo Guerrero-Lemus, Pilar Amo-Ochoa

**Affiliations:** † Dpto. de Química Inorgánica, Universidad Autónoma de Madrid, 28049 Madrid, Spain; ‡ Dpto. de Química, Universidad de La Laguna, 38207 San Cristóbal de La Laguna, Spain; § Dpto. Química, Universidad Autónoma de Madrid, 28049 Madrid, Spain; ∥ Institute for Advanced Research in Chemical Sciences (IAdChem), Universidad Autónoma de Madrid, 28049 Madrid, Spain; ⊥ Department of Organic and Inorganic Chemistry, University of the Basque Country UPV/EHU, 48080 Bilbao, Spain; # Dpto. Física Aplicada, Universidad Autónoma de Madrid, 28049 Madrid, Spain; ∇ Dpto. de Física, Universidad de La Laguna, 38207 San Cristóbal de La Laguna, Spain

## Abstract

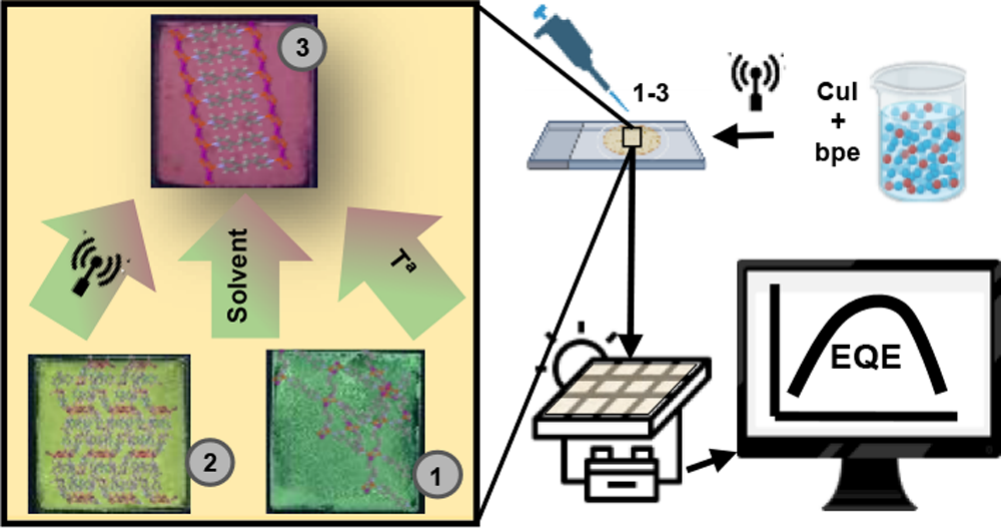

Downshifters refer to compounds with the capacity to absorb UV photons and transform them into visible light. The integration of such downshifters has the potential to improve the efficiency of commercial photovoltaic modules. Initially, costly lanthanide derivatives and organic fluorescent dyes were introduced, resulting in a heightened module efficiency. In a novel research direction guided by the same physicochemical principles, the utilization of copper(I) coordination compounds is proposed. This choice is motivated by its simpler and more economical synthesis, primarily due to copper being a more abundant and less toxic element. Our proposal involves employing 1,2-bis(4-pyridyl) ethane (*bpe*), an economically viable commercial ligand, in conjunction with CuI to synthesize coordination polymers: **[CuI(bpe)]**_***n***_**(1)**, **[Cu**_**3**_**I**_**3**_**(bpe)**_**3**_**]**_***n***_**(2),** and **[CuI(bpe)**_**0.5**_**]**_***n***_**(3)**. These polymers exhibit the ability to absorb UV photons and emit light within the green and orange spectra. To conduct external quantum efficiency studies, the compounds are dispersed on glass and then encapsulated with ethylene vinyl acetate through heating to 150 °C. Interestingly, during these procedural steps, the solvents and temperatures employed induce a phase transformation, which has been thoroughly examined through both experimental analysis and theoretical calculations. The outcomes of these studies reveal an enhancement in external quantum efficiency with [Cu_3_I_3_(bpe)_3_]_*n*_(**2**), at a cost significantly lower (between 340 and 350 times) than that associated with lanthanide DS complexes.

## Abstract 2

Copper(I) coordination compounds can be used as downshifters to enhance the efficiency of commercial photovoltaic modules, presenting an excellent alternative compared to more expensive and challenging to obtain materials such as those based on organic chromophores or lanthanide derivatives, among others. Dispersion and encapsulation processes, as well as the size of the dispersed particles, are key factors in understanding and improving EQE.

## 1 Introduction

The ligand 1,2-bis(4-pyridyl) ethane (*bpe*) has been extensively employed and investigated in the synthesis of novel compounds.^1,2^ Due to its ditopic nature and the high flexibility conferred by its −CH_2_–CH_2_– backbone, it has been utilized as a building block in conjunction with various metal centers (Cu(II), Co(II), Ni(II), Fe(II), etc.) to achieve a wide range of coordination polymers (CPs) exhibiting distinct dimensionalities (1D, 2D, and 3D)^3,4^ and properties.^5,6^ Numerous investigations with these types of CPs have reported porosity (MOFs),^7^ magnetism,^8−10^ and even luminescence^11^ depending on the chosen metal center.^12−18^ The majority of CP syntheses have involved solvothermal methods conducted under elevated pressures and temperatures to facilitate crystallization processes, in situ redox reactions, and the formation of novel coordination environments.^3,19^ While most research has focused on the structural attributes of these CPs,^20−22^ a limited number of studies have explored their captivating properties, such as high quantum yield luminescence, exemplified by compound {[Cu_2_I_2_(*bpe*)_2_]·Am}_*n*_^2,14^ (Am = aniline, or p-toluidine).^2,19^ Their remarkable optical properties suggest that CPs possessing similar characteristics could be of interest as downshifters, which are compounds capable of absorbing UV photons and converting them to visible light. Such downshifters could enhance the efficiency of commercial photovoltaic modules based on silicon solar cells.^23^ The limited efficiency of these modules (≈20%) is primarily attributed to the optical bandgap of silicon (*E*_gap_ ≈ 1.1 eV). This factor determines that the maximum absorbance values are within the visible range, while for IR and UV radiation, the absorbance and consequently the efficiency are considerably lower.^24,25^ To address this issue, lanthanide derivatives capable of absorbing UV radiation and emitting it in the enhanced visible range, were introduced approximately a decade ago, thereby enhancing module efficiency.^26−29^ However, lanthanides are scarce resources, found in low concentrations mixed with other minerals, and their extraction and refining processes are highly expensive and generate substantial amounts of toxic and radioactive waste.^30,31^ Moreover, the availability of lanthanides on a large scale is geographically limited, with China currently accounting for around 70% of their production. It is noteworthy that the most significant outcomes thus far have been achieved with a europium compound having the formula [Eu(tta)_3_(phen)] (phen: 1,10-phenanthroline, tta: 2-thenoyltrifluoroacetone), with an estimated cost of 6070 €/kg.^32,33^ Additionally, expensive commercially available organic fluorescent dyes like BASF LUMOGEN 570 F Violet (7000–9000 €/kg) have also been investigated and showed an increase an increase of over 10% in external quantum efficiency (EQE) for the 300–400 nm region.^34^ These factors underscore the necessity for exploring downshifters based on more abundant and less toxic elements such as copper.

Furthermore, in photovoltaic modules, solar cells are shielded from external factors by using a series of components, including front and rear encapsulates primarily composed of transparent organic polymers serving as sealing sheets. One widely used material is ethyl vinyl acetate (EVA), which, upon heating to 150 °C under vacuum treatment, forms a protective layer insulating the solar cells.^35^

The proposed novel and little explored idea involves incorporating copper(I) coordination compounds with the aforementioned properties into the photovoltaic module by dispersing them within the EVA layer.^34,36−39^ The process can be easily implemented on a large scale. In fact, a preliminary study with the CP [Cu(NH_2_MeIN)I]_*n*_ (NH_2_MeIN = methyl, 2-amino isonicotinate) showed promising results, with an increase over 0.015% in EQE in the UV region (300–400 nm) when adding 5% of the compound onto EVA.^39^ This opens the door to controlling other factors of the compound under investigation, such as the photoluminescent quantum yield, particle size, or thermal stability, aiming to achieve maximum efficiency at commercially competitive levels.

Specifically, we have evaluated the only two 2D CPs of the *bpe* ligand alone with CuI published to date whose chemical formulas are **[Cu**_**3**_**I**_**3**_**(*****bpe*****)**_**3**_**]**_***n***_**(2)**(1) and **[CuI(***bpe***)**_**0.5**_**]**_***n***_**(3)**,^19^ along with a new compound, **[CuI(*****bpe*****)]**_***n***_**(1),** developed by us and obtained by altering the previously employed reaction stoichiometry, temperature, and the use of KI, among others factors.^40^ The optical properties of these compounds have been thoroughly examined, indicating favorable characteristics for their potential utilization as downshifters. Compounds **1**–**3** absorb photons within the UV range and emit light in green (**1** and **2**) and orange regions (**3**). However, prior to conducting EQE studies on photovoltaic modules, it is necessary to manufacture dispersions of these materials and disperse them on the quartz (SiO_2_) surface, followed by an encapsulation process with EVA. These studies have revealed that compounds **1** and **2** undergo a phase transformation to **3** when they are dispersed by sonication in acetonitrile or dichloromethane solvents, or subjected to temperatures of 150 to 170 °C, respectively.^25,41−43^ This phase transformation is accompanied by a significant change in the emission color, transitioning from green to orange. In this work, these phase transitions are thoroughly analyzed, and their influence on the external quantum efficiency (EQE) results is discussed.

## 2 Materials and Methods

### 2.1 Synthesis

#### 2.1.1 Synthesis of **1** Polycrystals

First of all, *bpe* (0.22 g, 1.2 mmol) is dissolved in CH_3_CN (3 mL), under magnetic stirring (700 rpm) at room temperature. After that, another solution of CuI (0.12 g, 0.63 mmol) in CH_3_CN (7 mL) is added to the bpe solution. A pale yellow precipitate is formed immediately, and the reaction is allowed to stir at 25 °C for 30 min. The obtained precipitate is filtered off under vacuum and washed with CH_3_CN (4 mL). Finally, the precipitate is dried under vacuum for 5 h (234 mg, yield: 99%, based on CuI). Elemental analysis (%) calculated for [CuI(C_12_H_12_N_2_)]_*n*_: C, 38.47; H,3.23; N, 7.48. Experimental: C, 38.79; H,3.55; N, 7.48. IR (ν, cm^–1^): 3035 (w), 3024 (w), 2929 (w), 1605 (s), 1558 (m), 1494 (m), 1450 (m), 1417 (m), 1218 (s), 1072 (m), 1012 (m), 989 (m), 965 (m), 827 (s), 811 (m), 765 (w).

#### 2.1.2 Synthesis of **1** Single Crystals

Yellow single crystals are obtained in a test tube, after the slow addition at room temperature, and without stirring, of a solution of *bpe* (0.11g, 0.59 mmol) in CH_3_CN (2 mL), over a solution of CuI (0.055 g, 0.29 mmol) in CH_3_CN (4 mL). After 18 h, the yellow single crystals are collected by vacuum filtration, washed with CH_3_CN (4 mL), and dried under vacuum for 5 h (94.5 mg, yield: 87%, based on CuI). IR (ν, cm^–1^): 3035 (w), 3024 (w), 2946 (w), 1605 (s), 1557 (m), 1493 (m), 1452 (m), 1416 (s), 1217 (s), 1071(m), 1011(m), 989(w), 964(w), 828(s), 809(s), 764(w).

#### 2.1.3 Synthesis of **2**

This compound has been reproduced following the previous synthesis published by Lang and co-workers.^1^ A mixture of *bpe* (54 mg, 1 mmol) and CuI (57 mg, 1 mmol) both previously dissolved in CH_3_CN (6 mL) is stirred magnetically (700 rpm) under reflux at 90 °C for 48 h. The mixture is cooled to room temperature at a rate of 5 °C h^–1^ producing a yellow precipitate. The precipitate obtained is filtered off under vacuum, washed with CH_3_CN (2 × 2 mL), and dried under vacuum for 24 h (304 mg, yield: 27%, based on CuI). Elemental analysis (%) calcd for [Cu_3_I_3_(C_36_H_36_N_6_)]_*n*_: C, 38.51; H,3.23; N, 7.49. Experimental: C, 38.79; H, 3.55; N, 7.48. IR (ν, cm^–1^): 3039 (w), 3017 (w), 2923 (w), 1608 (s), 1557 (m), 1500 (m), 1422 (s), 1386 (w), 1344 (w), 1219 (m), 1124 (w), 1075 (m), 1018 (m), 827 (s).

#### 2.1.4 Synthesis of **3**(19)

This compound has been reproduced following the previous synthesis published by Ki et al.^19^ A KI-saturated solution (2 mL) containing CuI (0.19 g, 1 mmol) and CH_3_CN (2 mL) was added into a *bpe* (0.18g, 1 mmol) solution in methanol (2 mL). The precipitate obtained is filtered off under vacuum, washed with CH_3_CN (3 × 3 mL), and dried under vacuum for 12 h (339 mg, yield: 60%, based on CuI). Elemental analysis (%) calcd for [Cu_2_ I_2_ (C_12_ H_12_ N_2_)]_*n*_: C, 25.50; H,2.14; N, 4.96. Experimental: C, 25.51; H, 2.53; N, 4.98. IR (ν, cm^–1^): 3041(w), 1608(s), 1553(w), 1489(m), 1413(m), 1343(w), 1276(w), 1229(m), 1076(m), 1020(m), 807(s), 689(w), 672(w).

#### 2.1.5 Transformation to **3**(1)

The compounds **1** and **2** are transformed into **3**(19) when the temperature is raised to 150 and 170 °C, respectively. They also transform into compound **3** when it is in contact for a prolonged time with some organic solvents such as acetonitrile or dichloromethane.

#### 2.1.6 Preparation of Dispersions of **1**, **2**, and **3** @solvent-0.5 and 1% in Mass

To prepare the dispersions (0.5 and 1%) of **1** in MeOH/H_2_O (1:1 v/v), **2** in MeOH, and **3** in CH_3_CN, the calculated quantity of compound is dispersed in a given volume according to Table 3. The mixtures were stirred magnetically (700 rpm) for 10 min and then homogenized in an ultrasonic bath (25 °C, 40 kHz, 40% power) for 10 min. Next, 500 μL of suspensions is deposited on 2.5 × 2.5 cm quartz surfaces using the drop-casting technique. They are left to dry at room temperature until the respective solvents have evaporated.

### 2.2 Materials

The reagents and solvents were used without prior purification. Copper(I) iodide (CuI, 98%) was purchased from Sigma-Aldrich (CAS: 7681–65–4), and ligand 1,2-Bis(4-pyridyl)ethane (bpe, 98%) was purchased from Tokio Company International (TCI) (CAS: 4916–57–8). The solvent used both in the synthesis and in the preparation of the dispersions is acetonitrile (CH_3_CN), which was purchased from LabKem, with HPLC degree of purification (CAS: 75–05–8). The other two solvents used for the preparation of the dispersions are methanol (MeOH) and dichloromethane (CH_2_Cl_2_) obtained from Sigma-Aldrich and Thermo Scientific (CAS: 67–56–1, 75–09–2). The solvents used, isopropanol (C_3_H_8_O), N,N-dimethylformamide (DMF), and chloroform (CHCl_3_), were purchased from Scharlau (CAS: 67–63–0, 68–12–2, 67–66–3); trichlorethylene (C_2_HCl_3_) was purchased from Sigma-Aldrich (CAS: 79–01–6); acetone was purchased from Carlo Erba (CAS: 67–64–1); and dimethyl sulfoxide (DMSO) was purchased from PanReac (CAS: 67–68–5).

### 2.3 Methods and Equipment

Infrared spectra were obtained using a PerkinElmer 100 spectrophotometer with a Universal Attenuated Total Reflectance (ATR) sampling accessory. Elemental chemical analysis (AQE) was performed with a LECO CHNS-93217 elemental analyzer. Single crystal X-ray diffraction (XRD) has been performed using a Bruker Kappa Apex II Single Crystal diffractometer, equipped with a cryostat for data collection at low temperature or in an inert atmosphere, a kappa geometry goniometer, and a sealed molybdenum tube (λ*M*_o_ = 0.7107 Å). The powder X-ray diffraction data were done using a Diffractometer PANalytical X’Pert PRO with a θ/2θ scanning monochromator and X’Celerator fast detector and 1° primary monochromator for Kα1. The samples were performed by scanning θ, from 3 to 50°, with a time per increment of 1000 s and an angular increase of 0.0167.

Scanning electron microscopy (SEM) images were taken using a Philips XL 30 S-FEG electron microscope, applying an electron beam of 10.0 kV of potential and 300 μA of intensity, at a pressure of 10^–7^ Pa. Samples were metallized with a 15 nm thick Au layer, at a pressure of 10^–3^ Pa.

For the thermogravimetric analysis (TGA) measurement, a TA Instruments Q500 thermobalance oven was used with a platinum sample holder. The experiment was carried out under a N_2_ atmosphere at a flow rate of 90 mL min^–1^ and a heating rate of 10 °C min^–1^, in a temperature range of 25 to 1000 °C.

The external quantum efficiency (EQE) measurements have been carried out on a photovoltaic mini-module consisting of a p-type multicrystalline silicon solar cell (nontextured and with a SiNx antireflection coating optimized at 600 nm); this solar cell is encapsulated in standard solar glass and shows a conversion efficiency of 16%. A standard EQE configuration based on a 100 W Xe arc lamp, a monochromator, and a digital lock-in amplifier have been used, integrated into the commercial SPECLAB configuration of the Fraunhofer ISE laboratory (Germany). Samples have been measured within a defined area on top of the mini-module both before and after encapsulation to guarantee the reproducibility of the measurements. A Bentham DH-Si silicon photodiode, responsive within the 200–1100 nm range, served as the reference for obtaining consistent EQE values. The methodology followed adhered to a standard reference cell approach,^44^ where the input power is determined with the tabulated EQE of the Bentham DH-Si silicon photodiode. The EQE was obtained following *E*_c_, 1, where *A*_ref_ and *A*_test_ represent the reference cell and the testing device areas, respectively, ASR_ref_ is the absolute spectral response, *dI*_sc_(λ) represents the short circuit current values of the test device, and *dI*_sc,ref_(λ) is the reference cell, both measured at the same wavelength value (λ), and finally, *e*, *h*, and *c* represent the electron charge, the Planck’s constant, and the speed of light, respectively.^44^
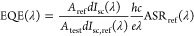
1Transmittance has been measured using an Agilent 8453 UltraViolet-Visible spectrophotometer, in the range of 0 to 1000 nm (λ).

For sonication in an ultrasonic bath, a Transsonic Digital S, Elma unit, is used at a power of 60% and a bath temperature of 25 °C.

To obtain the excitation and emission spectra, we positioned the powdered samples directly on quartz at 298 K and incident with the xenon lamp beam. The spectra were recorded in the spectral range 300–1000 nm using a Spex Fluorolog II equipped with a 450 W Xe lamp as an excitation source and two 0.22 m monochromators (Spex 1680). Emission is detected with a 950 V photomultiplier tube (PMT) operating in photon counting mode.

A 2-channel 300 MHz BRUKER AVANCE III-HD NANOBAY 300 MHz spectrometer equipped with a 5 mm BBO 1H/X probe, Z-gradient unit, and variable temperature unit was used to obtain the Nuclear Magnetic Resonance (RMN) spectra. Deuterated acetonitrile was used to perform the experiment.

Theoretical calculations were performed in the framework of the density functional theory (DFT) imposing periodic boundary conditions with the Vienna Ab initio simulation package (VASP). The electron density was expanded on a plane-wave basis until a cutoff value of 420 eV. We imposed a convergence criterion of 10–5 eV for the electronic density. The structures were fully optimized (both atomic positions and lattice vectors), and they were considered as converged when all the forces were lower than 0.01 eV/Å using the OPTPBE functional, which allows for the inclusion of weak interactions (i.e., van der Waals forces).

On top of the optimized structures, we have performed single point calculations with the Heyd–Scuseria–Ernzerhof (HSE) hybrid functional in order to reproduce accurately the band structure.

Reciprocal space was sampled using the Monkhorst–Pack scheme, using different *K*-meshes depending on the size of the cell in the real space, with values from only the Γ-point (**1** and **2**) to 5–3–3 (**3**).

## 3 Discussion

Compound **1** is synthesized by conducting the reaction at 25 °C in acetonitrile using CuI and 1,2-bis(4-pyridyl)ethane (*bpe*) as reagents in a 1:2 stoichiometric ratio. The mixture of both reagents under magnetic stirring promptly produced a precipitate. To enhance the final yield (99%), the stirring is prolonged for 30 min. Subsequently, the precipitate is separated via vacuum filtration and finally washed with cold acetonitrile. High-quality single crystals suitable for single crystal X-ray diffraction are obtained by carefully layering the two reagents, dissolved in acetonitrile, on top of each other within a test tube, allowing for slow diffusion (Figure S1).

The simulated theoretical powder X-ray diffractogram matches completely with the powder X-ray diffractogram obtained from **1** polycrystals, confirming the correspondence between both phases (Figure S2). Additionally, its elemental analysis confirms its empirical formula as [CuI(*bpe*)]. Finally, the infrared spectrum exhibits slight variations compared to that of *bpe*, specifically, a shift toward lower wavelengths in the bands associated with C–H tension, and higher wavelengths in the bands associated with C=C tension and CH flexion, indicating the binding of the copper(I) ion to the pyrazine nitrogen atom (Figure S3). Compounds **2** and **3** have been synthesized following the methods previously published by Li and Ki et al.,^1,19^ employing a 1:1 stoichiometric ratio of the *bpe* and CuI. The reaction for compound **2** is carried out in acetonitrile under solvothermal conditions, while for compound **3**, a KI-saturated acetonitrile/methanol solution is used. Additionally, compound **3** can be generated by chemical and thermal transformation of **1** and **2**.

### 3.1 Single-Crystal X-ray Diffraction Studies

**Figure 1:**
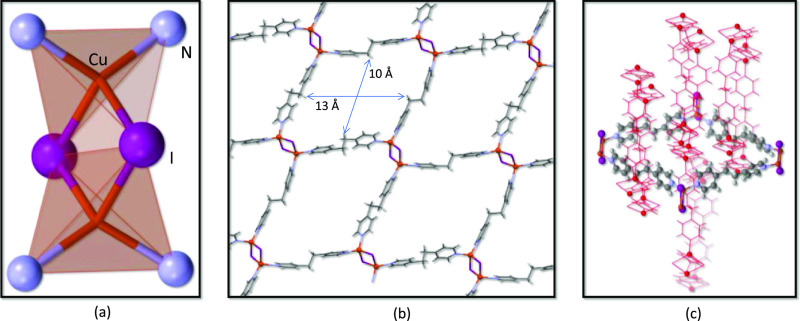
(a) [Cu_2_I_2_] dinuclear cluster in **1** with an atom numbering scheme. (b) *bpe* ligands, in an *anti*-configuration, linking the [Cu_2_I_2_] clusters to generate a 2D subnetwork. (c) Perpendicularly arranged 2D subnetworks interpenetrating among them with three subnetworks, depicted in red, going through every cavity of a perpendicularly arranged fourth.

Compound **1** crystallizes in the tetragonal system with the *P*4_3_2_1_2 space group. Its structure reveals Cu(I) ions forming edge-sharing tetrahedral [N_2_CuI_2_CuN_2_] dinuclear clusters (Figures 1a and S4), which are held together by four μ-bpe-κ*N*:κ*N′* bridging ligands with an *anti-conformation* to give rise to 2D sheets containing huge cavities (10 × 13 Å^2^, Figure 1b). The double values for each of these distances come from the fact that the metal centers placed in the sheets grow with different orientations. Considering the Cu_2_I_2_ dinuclear cluster as the node and the *bpe* ligands as the linkers, the topology of the 2D array can be described as a four-connected (4-c) herringbone^45^ 1a two-dimensional network according to the classification of coordination polymers of copper(I) halides published by Graham.^46^ Interestingly, there are two families of these 2D sheets, crystallographically independent but chemically equivalents, which only differ in the orientation of their planes, which are perpendicularly arranged and entangled among them (Figure 1c). The entanglement implies that every cavity present in the 2D sheet has three perpendicular sheets going through it and leading to a nonporous final structure. Inside every sheet, we can distinguish two copper···copper distances, 2.943 and 3.044 Å for copper atoms bridged by double μ*-*I^–^ anions and 13.369 and 13.412 Å for copper atoms bridged by the μ-bpe-κ*N*:κ*N′* ligands. Bond distances and angles are provided in Table S2 and are similar to those found in analogous compounds.^19,1,46,47^

**Figure 2:**
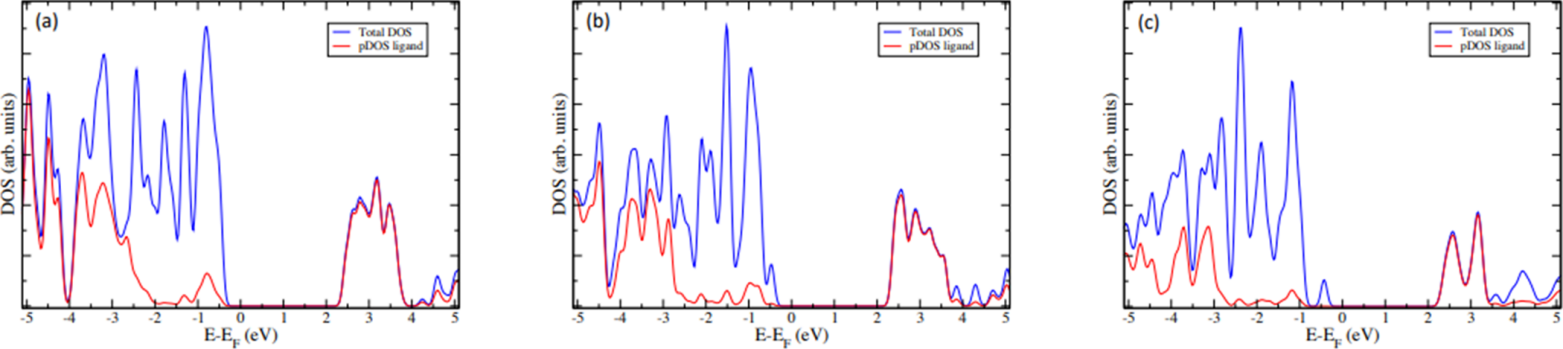
Density of States (DOS) of compounds **1**(a), **2**(b), and **3**(c) (blue) and their projection of the atoms of the ligands (red).

The structure of **2**, a polymorph of **1**, has been previously described,^1^ but for comparative purposes, a brief description is provided herein. There are tetrameric Cu_4_I_4_N_6_ and monomeric CuIN_3_ entities that are held together by bridging *bpe* ligands with *anti* and *gauche* conformations (Figure 2e,f). The *anti*-conformation appears for bridges connecting the tetrameric units among them and with the monomeric unit, whereas the gauche conformation is present when connecting adjacent monomeric units between them. Another difference is that although the resulting coordination bond sustained network is again a 2D one, it is clearly nonplanar. Although all of the tetrameric fragments, within the 2D sheet, lie in the same plane, the CuIN_3_ metal center is displaced from this mean plane by 4.9 Å. Finally, compound **3**, which contains half of the *bpe* ligands compared to **1** and **2**, presents a 2D network that was previously reported by Li and co-workers.^48^ In this case, a linear inorganic core of double ladder-like (CuI)_*n*_ is present connected by *bpe* ligands to adjacent 1D inorganic cores above and below to generate a coordination bond based 2D planar network (Figure 2d). These sheets are piled up in an AB staggered order (Figure 2c). The connectivity of the iodides also provides a clear distinction between these compounds with only μ_2_ bridges in compound **1**, a mixture of μ_2_-, μ_3_-, and terminal iodides in compound **2**, and only μ_3_ bridges in compound **3**. There are also some disparities on the Cu···Cu (2.94–3.10 for **1**, 2.70–3.49 for **2**, and 2.79 for **3**), Cu–I (2.65–2.66 Å for **1**, 2.60–2.74 Å for **2**, and 2.63–2.73 Å for **3**), and Cu–N (2.05–2.06 Å for **1**, 2.02–2.09 Å for **2**, and 2.04 Å for **3**) distances, although they still fall within reasonable ranges for compounds of this nature.^19,49^

### 3.2 Photoluminescent Studies

A qualitative investigation reveals that compounds **1** and **2** exhibit a pronounced green emission, while compound **3** exhibits an orange emission when excited at 365 nm at 25 °C (Figure S5). This observation aligns with the quantitative data obtained in the solid state at 25 °C. Upon exciting **1** and **2** at 396 nm, intense emission bands at 496 and 500 nm are observed, respectively (Figures S6 and S7). In the case of the previously published compound **2**, photoluminescence studies were conducted using an excitation wavelength of 285 nm, which explains the observed discrepancy in our case where the study was performed with an excitation wavelength of 396 nm.^1^

In the case of compound **3**, after being excited at 360 nm, the emission bands are obtained at 430 and 577 nm (Figure S8). The previously published photoluminescent results on this compound show discrepancies, as they indicated the presence of a single band around 440 nm.^19^ However, the researchers characterized the compound only through powder X-ray diffraction, lacking elemental analysis or IR data that could confirm the presence of impurities, and there are no images showing its emission.

In all three cases, a red shift with respect to the ligand (λ_exc_ 353 nm, λ_em_ = 422 nm (Figure S9) is observed.^1^ In the case of compound **2**, the observed peaks are assigned to the two structural units, the mononuclear [CuI], and the chairlike [Cu_4_(μ-I)_2_(μ_3_-I)_2_] tetrameric subunits.^1,19^ The emission bands are a consequence of the combination of the ^3^CC of the excited state containing mixed metal halide charge transfer (XMCT) band and the d-s transitions due to Cu(I)–Cu(I) interactions, which are in agreement with copper(I) coordination polymers of N-containing ligands reported by others.^2^ (Table 1).

**Table 1:** Emission Wavelength Data of Compounds **1–3**, Unheated, and **1** and **2** Subjected to Heating (150 and 170 °C Respectively) for 15 min

compound	emission(nm)	color
**1** λ_exc_ = 396 nm.	496	green
**2** λ_exc_ = 396 nm	418 and 500	green
**3** λ_exc_ = 360 nm	430 and 577	orange
**1** + 150 °Cλ_exc_ = 375 nm	423 and 585	orange
**2** + 150 °Cλ_exc_ = 352 nm	418 and 500	green
**2** + 170 °C λ_exc_ = 352 nm	423 and 582	orange

Analyzing the density of states (DOS) projected onto the distinct chemical elements within the unit cell (pDOS) enables the characterization of electronic transitions crucial for potential applications of the investigated compounds as downshifters. In each of the three cases, states with high energies (Fermi level and states immediately below this energy) primarily localize around the CuI units, while the lowest unoccupied levels are situated within the ligand, as depicted in Figure 2. Consequently, the transitions of significance are metal-to-ligand charge transfer transitions.

**Figure 3:**
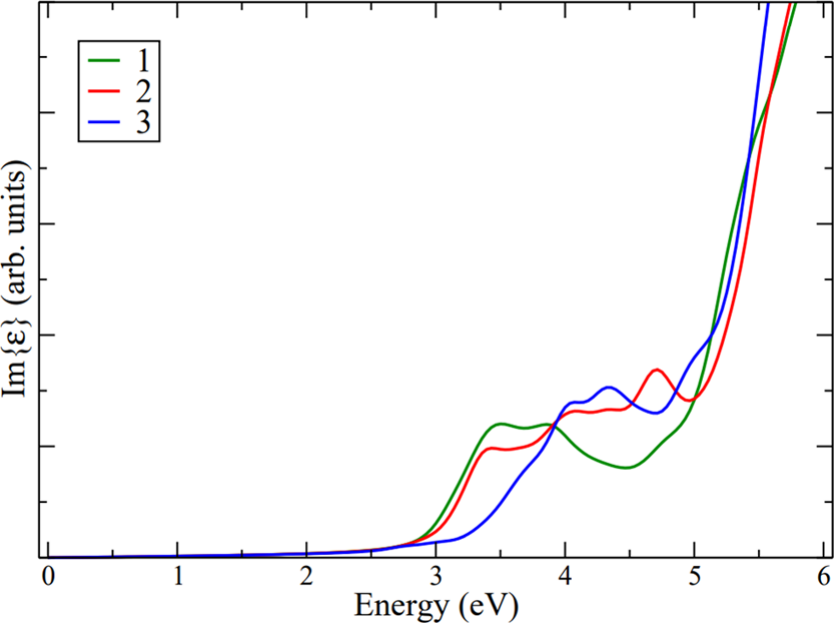
Imaginary part of the dielectric function of **1** (green), **2** (red), and **3** (blue).

The calculated imaginary part of the dielectric function (Figure 3), which is proportional to the material’s absorption, exhibits excellent agreement with the experimental spectra. In all the cases, absorptions begin at energies higher than the fundamental (HOMO–LUMO) transition extracted from the DOS. Two factors contribute to this phenomenon: (i) the higher value of the DOS at energies below the HOMO (absorption is proportional to the DOS, following Fermi’s golden rule); (ii) the potential inactivity (darkness) of the fundamental transition due to the symmetry of the involved states.

Upon electron excitation, the hole generated is filled by electrons from the HOMO. When the material returns to the ground state, the transition occurs at a lower energy level. Following Kasha’s rule, the radiative deexcitation is observed from the lowest state of a given multiplicity. In this case, the theoretical HOMO–LUMO gaps of compounds **1**–**3** are able to provide an explanation of the emission colors. While **1** and **2** have similar band gap values (2.9 and 2.8 eV respectively), **3** exhibits the lowest gap, with a value of 2.7 eV. These data are in good agreement with those obtained experimentally at room temperature through the Kubelka–Munk function (Figure S10).

### 3.3 Thermal Phase Transformation

The investigation into the thermal stability of compounds **1** and **2** reveals an initial mass loss around 150 and 170 °C, respectively, likely associated with the partial loss of the ligand (*bpe*). Subsequent stages occur between 180 and 200 °C, respectively, indicating complete ligand loss, until their total decomposition around 495 and 440 °C, respectively (Figure S11). The decomposition temperature of compound **3**, around 280 °C, has been previously studied.^19^

Considering the thermal stability, both compounds were heated for 15 min at 150 and 170 °C, respectively, observing a qualitative change in the emission from the initial green to orange (Figure S12). Additionally, emission spectra were obtained for both compounds after heating, showing in both cases, the orange emission characteristic of **3** (Figures S13 and S14). The X-ray powder diffraction patterns and IR spectra of compounds **1** and **2** after heating at 150 and 170 °C, respectively, for 15 min also corroborate the phase transition to compound **3** by completely matching (Figures S15–17).

In our opinion, this transformation is clearly due to an entropic effect that facilitates the release of some of the *bpe* ligands in the gas phase or solution, where their entropy is relatively high. However, there is doubt whether these transitions involve a previous transformation among polymorphs **1** and **2**. The possible routes on these transitions could be summarized as follows: (a) **1** → **2** → **3**, (b) **2** → **1** → **3,** or (c) **1** → **3/2** → **3**. In fact, the significantly different density of the polymorphic compounds **1** (1.94 g/cm^3^) and **2** (1.88 g/cm^3^) can provide a clue on their thermodynamic stability; in other words, which one is the thermodynamically stable phase and which one is the metastable phase. Usually, the more dense phase corresponds to the more stable phase, and in this case, it also agrees with the phase, with the structure showing a smaller dispersion on the metal center coordination environments and the *bpe* ligand conformations. It seems to preclude option b (**2** → **1** → **3**), but it does not allow us to distinguish between options a (**1** → **2** → **3**) and c (**1** → **3/2** → **3**).

**Figure 4:**
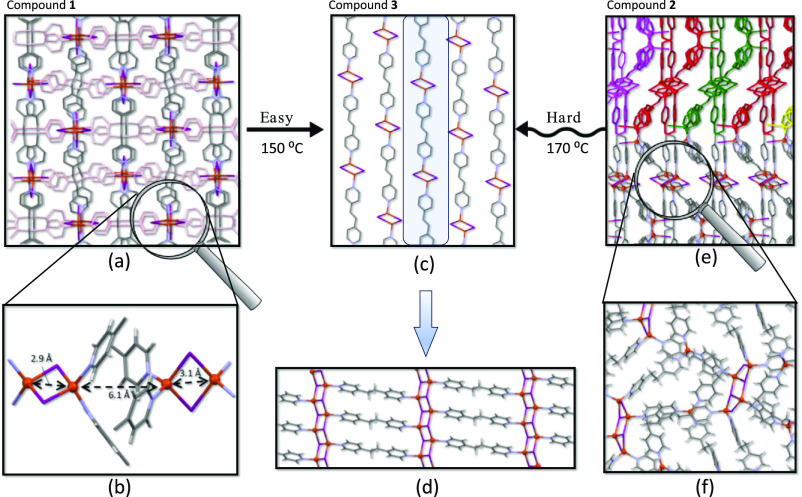
(a) Crystal packing of **1** along the crystallographic *c* axis showing the similarity to **3** when half of the *bpe* ligands are removed (depicted in pale pink). (b) Details of the close approach between the Cu_2_I_2_ dimeric subunits belonging to the entangles 2D sheets in **1**. (c) Crystal packing of **3** along the crystallographic *a* axis. (d) Details of the 2D network present in **3**. (e) Crystal packing of **2** along the crystallographic [−102] direction; each 2D sheet is partially shown in different colors to provide a visual guide to distinguish the 2D nature of this compound. (f) Insight into the 2D sheets present in **2**.

The TGA/DSC, X-ray powder diffraction, and IR results collectively indicate that the transition toward **3** takes place at 150 °C for compound **1** and at 170 °C for compound **2** with no discernible transformation between the polymorphs (compounds **1** and **2**). Therefore, the transition takes place directly from **1** and **2** to **3** (option c). The varying transition temperatures can be rationalized on the basis that a smaller rearrangement is required for the transformation, necessitating a lower temperature. In Figure 4, an insightful representation illustrates that despite the significant difference in crystal structure between **1** and **3** (entangled 2D sheets vs parallel stacked 2D sheets), the position of the heavier atoms (copper and iodine) is relatively similar in both structures, leading to a lower transition temperature (150 °C). In the case of **2**, it appears to be closer to compound **3** due to its parallel-aligned 2D sheets, but the placement of the heavier copper and iodine atoms significantly differs from the structure of **3**. Across these three compounds, the similarity in the placement of the heavier atoms emerges as more crucial than topological similarities in determining the transition temperature.

### 3.4 Chemical Phase Transformation

**Figure 5:**
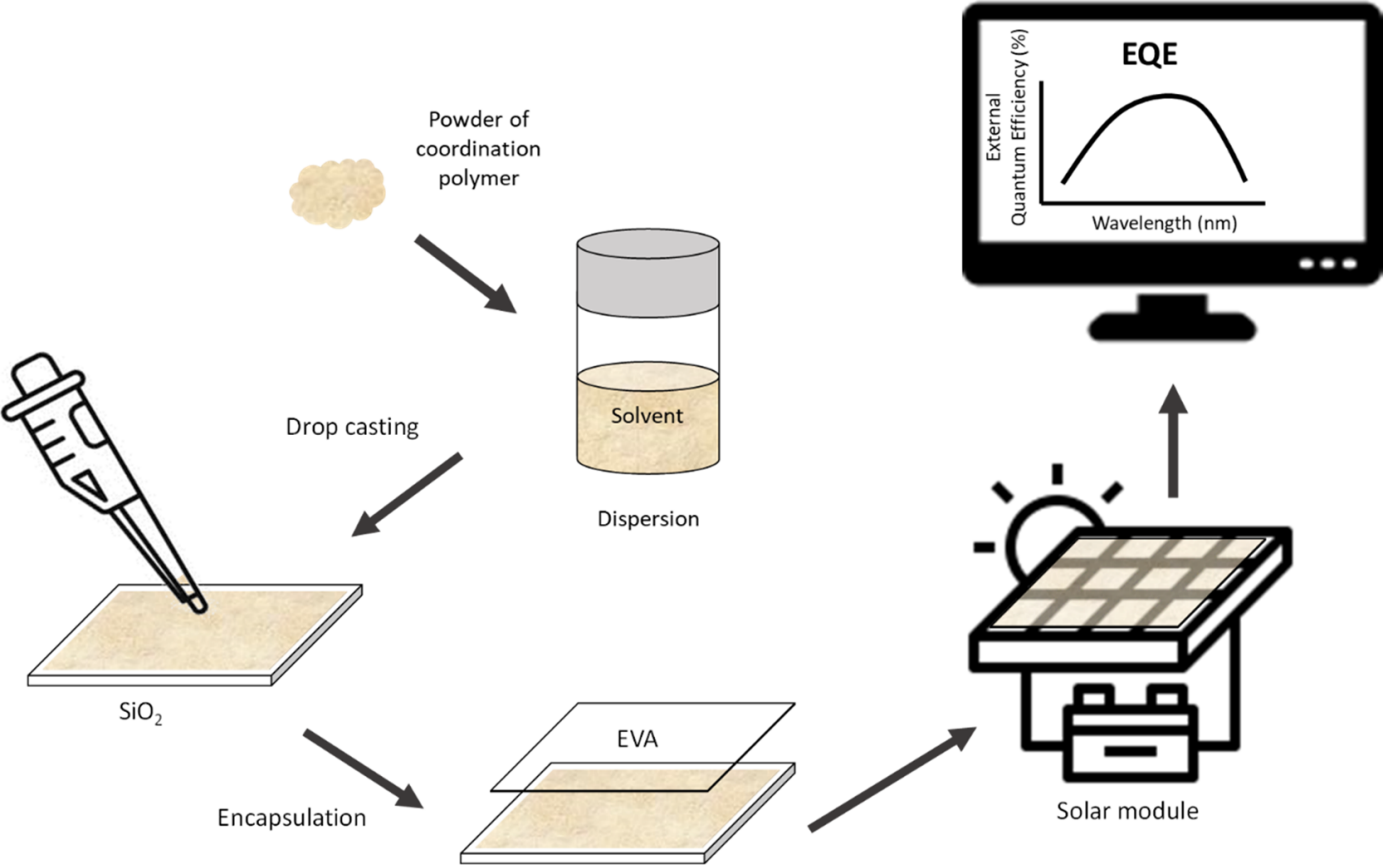
Schematic representation of the study of the EQE, started with the preparation of the dispersions, drop-casting of the compounds on quartz, and encapsulation with EVA in the commercial module.

In the course of external quantum efficiency (EQE) studies for these compounds on silicon photovoltaic modules, it is essential to effectively disperse the compounds on a quartz surface, (glass is the material used as a front cover in the commercial photovoltaic modules)^50^ that will later be covered in turn by a commercial encapsulant, the organic polymer ethylene-vinyl acetate (EVA) (Figure 5). The encapsulation process involves heating the sample up to 150 °C under vacuum for 1 h so that the encapsulant perfectly seals the solar cells, protecting them from external factors such as dust, etc.^51^

To obtain an adequate dispersion of the compounds by drop-casting on the quartz surfaces (2.5 × 2.5 cm), generating a thin and homogeneous layer, its stability in different solvents (CH_2_Cl_2_, MeOH, H_2_O, and CH_3_CN) was previously checked by adding 10 mg of each compound in 2 mL of the selected solvent and stirring magnetically for 10 min. The results (Tables S3 and S4) show that **1** and **2** (despite the fact that previous studies carried out on **2** have reported that it is not soluble in most common organic solvents such as CH_2_Cl_2_ and CH_3_CN)^1^ present a chemical transformation to compound **3** in the presence of acetonitrile and dichloromethane (Figure S18). This transformation has been followed by ^1^H NMR in deuterated acetonitrile at 25 °C for 35 min, observing an increase in the signal’s intensity of the corresponding *bpe* ligand, indicative of a decoordination of the ligand in the solvent. Additionally, a change in the initial emission of the compound (green) to orange has also been observed (Figures S19 and S20). Therefore, none of them can be used as dispersing agents.

### 3.5 Theoretical Calculations

To adequately understand both the thermal and chemical phase transformations to compound **3**, we used theoretical calculations. For this, we have carried out full geometry optimizations of the three compounds, including relaxation of the lattice constants (the final unit cells are very similar to the experimental ones, with discrepancies lower than 5%).

As shown in Table 2, DFT energies show that relative stability **3** compared with **1** and **2** strongly depends on the chemical environment of the ligand: in the absence of (stabilizing) interactions with the medium (such as intermolecular interactions) **3** is less stable. However, when these interactions are considered (in this study as the ligand in the condensed phase), **3** becomes the most stable phase. This is in good agreement with the experimental results, where **3** is produced when a solvent that can stabilize the ligand interacts with **1** or **2**. At high temperatures, entropic effects play an important role, overtaking the enthalpic effects and allowing for the formation of **3**. This process is irreversible since due to the stiffness of the structure and the size of the ligand, it is not able to enter again in the crystal to form **1** or **2**.

**Table 2:** Relative Energies of Compounds **1–3** (eV)

compound	relative energy (eV)
**1**	0.00
**2**	0.50
**3**^a^	11.15
**3**^b^	–3.75

a Lost ligand calculated in the gas phase.

b Lost ligand calculated in condensed phase.

### 3.6 External Quantum Efficiency Studies

For an accurate mean calculation of the EQE, it is crucial to control the various parameters. These include the amount of compound dispersed on the surface (0.5 and 1%), which is related to transmittance, and the solvent used in the dispersion, chosen to prevent any chemical transformation (MeOH/H_2_O for compound **1**, MeOH for compound **2,** and CH_3_CN in the case of **3**) (Table 3). Additionally, their thermal stability (as previously described) and particle size (Table 3) are important considerations.

**Table 3:** Summary of Relevant Parameters of Compounds **1–3**

compound@%	mass (mg)	particle size (μm)	solvent
**1**@0.5%	2.1	(*y*):0.77 ± 0.28	MeOH/H_2_O
		(*x*): 19 ± 7	
**1**@1%	4.0	(*y*):0.81 ± 0.28	MeOH/H_2_O
		(*x*): 18 ± 5	
**2**@0.5%	1.8	(*y*):6.84 ± 3.62	MeOH
		(*x*): 35 ± 7	
**2**@1%	3.7	(*y*):4.98 ± 2.39	MeOH
		(*x*): 43 ± 9	
**3**@0.5%	2.2	(*y*):0.41 ± 0.10	CH_3_CN
		(*x*):1.31 ± 0.42	
**3**@1%	4.1	(*y*):0.51 ± 0.16	CH_3_CN
		(*x*):1.54 ± 0.89	

In the process for preparing 2.5 × 2.5 cm quartz surfaces, 500 μL of the respective suspensions is deposited by drop-casting and left to evaporate at room temperature. The particle size observed by scanning electron microscopy (SEM) on the studied surfaces shows that compound **2** has dimensions between 5 and 7 and 35–43 μm (width × length), while for compounds **1** and **3, they** are between 0.4 and 0.8 and 1.3–19 μm (width × length; Table 3 and Figures S21 and 22).

**Figure 6:**
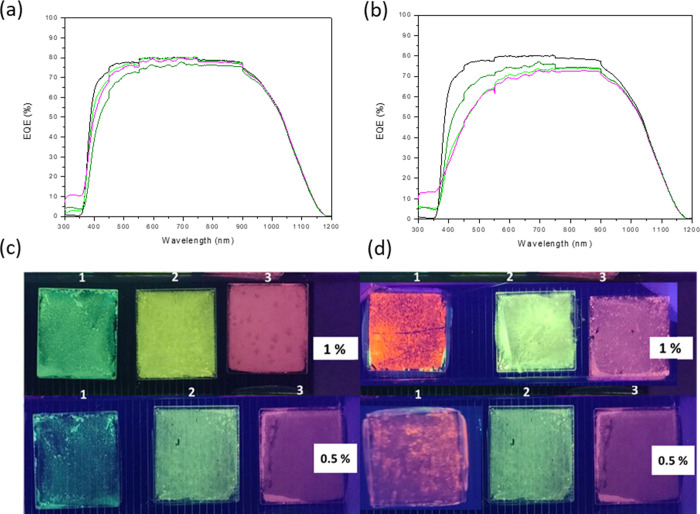
EQE compounds 0.5% (a) and 1% (b) deposition after encapsulation: mini-module (black), **1**@0.5 and 1% (light green), **2**@0.5 and 1% (pink), and **3**@0.5 and 1% (dark green). Compounds **(1–3)** on quartz before (c) and after (d) encapsulation in a photovoltaic mini-module emission under UV light (λ = 365 nm).

The examination of external quantum efficiency (EQE) involves placing the quartz surface containing the dispersed compound (0.5 and 1%) onto a multicrystalline silicon minimodule (Figure 6c). Subsequently, the composition is covered with commercial EVA and encapsulated by vacuum at 150 °C for 1 h (Figure 6d). EQE results indicate that compound **2** exhibits the highest efficiency, and this efficiency increases with the quantity of sample deposited. Specifically, samples **2**@0.5 and **2**@1% show an increase in EQE in the UV (300–350 nm) region of approximately 11.1 and 13.3%, respectively (Table 4, Figure 6a,b). However, despite these improvements, their integrated EQE versus wavelength across the entire measured range (300–1200 nm) remains slightly smaller than that of the mini-module (m-m) (520.23 vs 526.69, respectively) (Table S5). This observation, along with the decrease in transmittance with the increase in the amount of dispersed compound 2 (Figure S23), indicates the need for further enhancements of EQE within the visible range. This primarily involves the combined optimization of the compound concentration and thickness of the luminescent film to ensure the highest transparency of the film in the visible spectral range. Additionally, the study of the EQE before the encapsulation process (Figure S24) allows us to verify that compound **2** again presents a higher EQE in the UV with respect to compound **1**. Taking into account that the particle size of compound **2** is approximately 9–10 orders of magnitude larger than that of compound **1** (both emitting in green), the size factor may also play a relevant role in enhancing efficiency. After the encapsulation process at 150 °C for 1 h, as expected, compound **1** has undergone a structural transformation to compound **3** (Figure 6), which is stable at least for 2 months, as confirmed by its powder X-ray diffraction (Figure S25), while compound **2** remains unchanged (Figure 6d).

**Table 4:** Price of Compounds **1**, **2**, BASF LUMOGEN 570 F Violet, and [Eu(tta)_3_(phen)]^a^

**1** (€/kg)	**2** (€/kg)	[Eu(tta)_3_(phen)] (€/kg)	BASF LUMOGEN 570 F violet (€/kg)^33^	**1**@0.5% MeOH/H_2_O (€/m^2^)	**1**@1% MeOH/H_2_O (€/m^2^)	**2**@1% MeOH/H_2_O (€/m^2^)	**2**@0.5% MeOH/H_2_O (€/m^2^)
22.29	19.66	6070	7000–9000	0.017	0.009	0.016	0.008

a Prices of the precursors and reactives: https://www.made-in-china.com/.

In our comparative analysis, this study also explored the EQE of the recently published compound [Eu(tta)_3_(phen)] dispersed in 5% EVA ([Eu(tta)_3_(phen)]@EVA-5%) under conditions similar to those described for compounds **1**–**3**. This particular compound enhances the EQE by approximately 8% in the 300 to 400 nm region (Figure S25). As previously mentioned, compound **2** at lower concentrations (0.5 and 1%) increases the EQE in the UV region between 3 and 5% more. Furthermore, if we talk in terms of the price of the material, the europium compound is on the order of 350 times more expensive than compound **2** (Table 4).

## 4 Conclusions

The primary goal of the research is to enhance the external quantum efficiency (EQE) of the commercial photovoltaic modules, offering a more economical alternative to previously explored materials based on lanthanides or organic dyes chromophores among others.

The selected copper(I) coordination polymers **1**–**3** present an intense emission in the visible region of the spectrum (between 418 and 585 nm) after absorbing ultraviolet radiation (between 300 and 395 nm) through a downshifting mechanism. The luminescent properties exhibited by the compounds combined with the cost-effectiveness of the resulting products (Table 4) position them as potentially valuable materials for use as downshifters in photovoltaic modules.

The study of EQE involves producing various photoluminescent films of these compounds with concentrations of 0.5% and 1 wt % using the drop-casting technique followed by correct encapsulation on a multicrystalline silicon minimodule. Our findings highlight that the dispersion and encapsulation processes associated with the generation of commercial photovoltaic modules that involve the use of organic solvents and high temperatures can induce chemical transformations in the selected compounds. In this research, compound **1** undergoes a chemical transformation due to its structural characteristics, as explained through a structural and theoretical study.

The EQE measurements of the minimodule with the encapsulated samples reveal that all luminescent films produce an increase in the external quantum efficiency of the minimodule in the ultraviolet region of the measured spectrum (300–350 nm), achieving an increment of 13.3% for **2** at 1% of concentration. This result surpasses the 8% increase observed in the study with the [Eu(tta)_3_(phen)] complex, leading to a cost reduction from 6070 to 19.66 €/kg (Table 4). The research also establishes that the particle size and the amount of compound are crucial factors in increasing EQE.

These compounds clearly introduce significant enhancement in the UV region, providing cell protection against higher energy radiation. However, in the visible spectrum, we have not managed to improve the efficiency compared to that of a cell without the active compound. This deterioration in the visible region is attributed to a slight decrease in the encapsulated transmittance. Nevertheless, the results encourage us to continue working on these promising and efficient compounds in the UV region as improvements in encapsulation transparency alone could yield positive effects across the entire spectrum.

## Supporting Information Available

Synthesis, single-crystal X-ray diffraction studies, photoluminescent studies, thermal phase transformation, chemical phase transformation; morphological study by SEM, and EQE studies (PDF)

## Abreviations

BPE: 1,2-bis(4-pyridyl) ethane
EQE: external quantum efficiency
EVA: ethylene vinyl acetate
ATR: attenuated total reflectance
AQE: elemental chemical analysis
XRD: single crystal X-ray diffraction
SEM: scanning electron microscopy
TGA: thermogravimetric analysis
RMN: nuclear magnetic resonance
DFT: density functional theory
VASO: Vienna ab initio simulation package
