# Supplementary material for: Determining Factors to Understand the External Quantum Efficiency Values: Study Carried Out with Copper(I)-I and 1,2-Bis(4-pyridyl)ethane Coordination Polymers as Downshifters in Photovoltaic Modules
Source: Inorg Chem. 2024 Mar 1;63(10):4646–56. doi: 10.1021/acs.inorgchem.3c04232 (PMC10934813; doi:10.1021/acs.inorgchem.3c04232)
Supplement: Supplementary file 1 — ic3c04232_si_001.pdf [file ic3c04232_si_001.pdf]

Supporting Information

Determining Factors to understand the external quantum efficiency values. Study carried out with Copper(I)-I and 1,2-bis(4-pyridyl)ethane coordination polymers as downshifters in photovoltaic modules

*Andrea García-Hernán[α], Gabriela Brito-Santos[β], Elena de la Rubia[α], Fernando Aguilar-Galindo[γ,δ], Oscar Castillo[χ], Ginés Lifante-Pedrola[ε], Joaquín Sanchiz[β], Ricardo Guerrero-Lemus[ϕ], Pilar Amo-Ochoa[α,δ]**

[α] *Dpto. de Química Inorgánica. Universidad Autónoma de Madrid, 28049 Madrid, Spain.*

[β] *Dpto. de Química Universidad de La Laguna. 38207 San Cristóbal de La Laguna. Spain.*

[γ] *Dpto. Química Universidad Autónoma de Madrid, 28049 Madrid, Spain*

[δ]*Institute for Advanced Research in Chemical Sciences (IAdChem). Universidad Autónoma de Madrid, 28049 Madrid, Spain*

[χ]*Department of Organic and Inorganic Chemistry, University of the Basque Country UPV/EHU, 48080 Bilbao, Spain*

[ε]*Dpto. Física Aplicada. Universidad Autónoma de Madrid, 28049 Madrid, Spain*

[ϕ]*Dpto. de Física. Universidad de La Laguna. 38207 San Cristóbal de La Laguna. Spain.*

E-mail: pilar.amo@uam.es

Synthesis

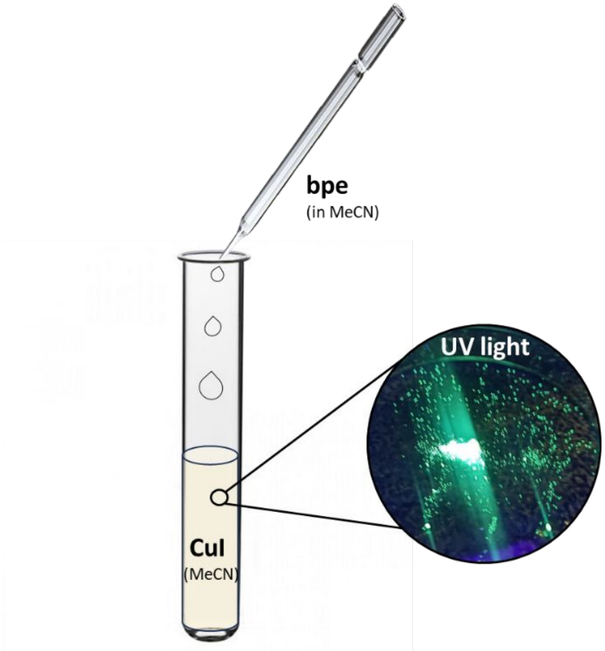

Figure S1. Crystallization scheme of **1**: The two reagents, dissolved in acetonitrile, are carefully layered on top of each other by dropwise addition within a test tube.

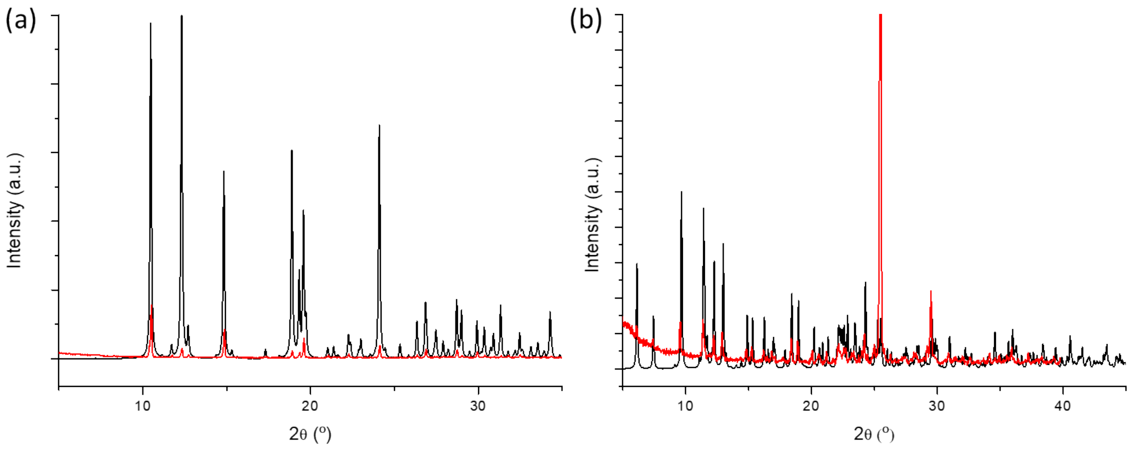

Figure S2. X-ray powder diffractogram at 25 ^{0}C of compounds (a) **1** and (b) **2**. The simulated powder pattern is shown in black and the experimental powder pattern in red.

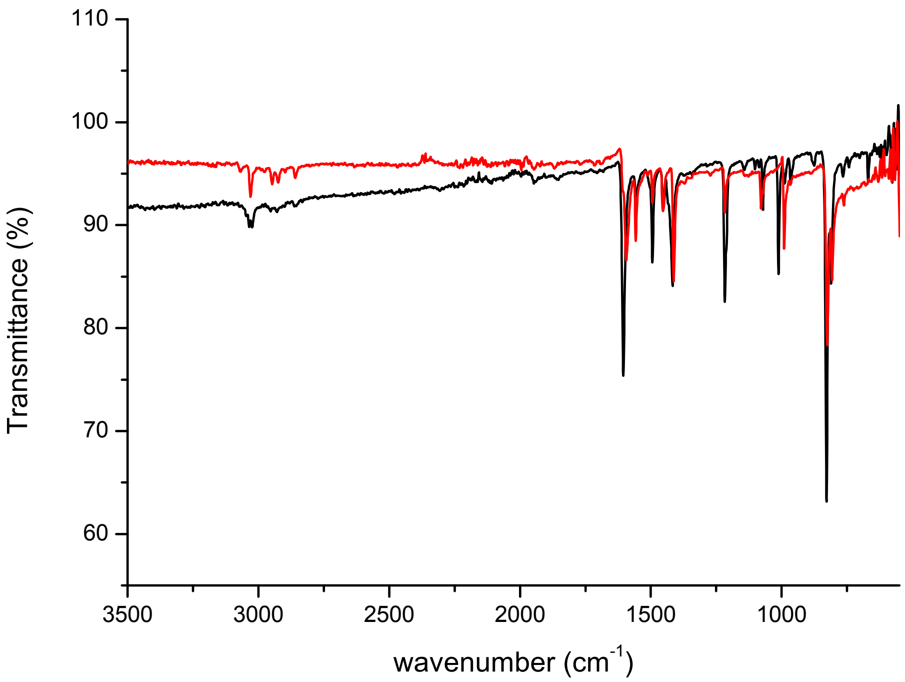

Figure S3. Infrared spectrum of **1** (black line) compared to the bpe ligand (red line).

3.1. Single-crystal X ray diffraction studies

Table S1. Crystal data, data collection, and structure refinement of compound 1

Empirical formula	$C_{12}H_{12}CuIN_2$
M/gmol^{-1}	374.69
Temperature (K)	298
λ/Å	1.54184
Crystal system, space group	Tetragonal, $P4_32_12$
a, b, c(Å)	16.8810(1), 16.8810(1), 17.9839(3)
α,β,γ (°)	90 90 90
V (Å^3)	5124.84(10)
Z	16
D_{calc}/gcm^{-3}	1.942
μ / (mm^{-1})	21.106
Theta range	3.5730 - 77.6120
No. of measured, independent and observed [I> 2s(I)] reflections	28046, 5390, 5227
R_{int}	0.0208
$R_1[I> 2\sigma(I)]^a$	0.0216
w$R_2[I > 2\sigma(I)]^b$	0.0545
GOF on $F^{2\ c}$	1.040

$^aR_1 = [\sum(\|F_o| - |F_c\|)/\sum|F_o|]$. $^b wR_2 = [\sum[w(F_o^2 - F_c^2)^2]/\sum[w(F_o^2)^2]]^{1/2}$

^{c}Goodness-of-fit $GOF = [\Sigma\ [w(F_o^2–F_c^2)^2] / (n–p)]^{1/2}$.

Table S2. Selected bond distances and angles in **1**.

Atoms 1,2	d 1,2 (Å)	Atoms 1,2	d 1,2 (Å)
Cu1—Cu1^i	3.0993(0)	I1—I2	4.3165(0)
Cu1—I2	2.6537(0)	Cu2—Cu2^{iii}	2.9427(0)
Cu1—I1	2.6603(0)	I3—I3^{iii}	4.4144(0)
Cu2—I3	2.6469(0)	Cu2—I3^{iii}	2.6599(0)
Cu1—N2^{ii}	2.0643(0)	Cu2—N4	2.0524(0)
Cu1—N1	2.0504(0)	Cu2—N3	2.058()

Atoms 1,2,3	Angle 1,2,3 (°)	Atoms 1,2,3	Angle 1,2,3 (°)
Cu2—I3—Cu2^{iii}	67.353(0)	Cu1^i—I2—Cu1	71.459(0)
N3—Cu2—N4	116.368(0)	I1—Cu1—N1	107.33()
I3^{iii}—Cu2—N3	103.958(0)	N1—Cu1—N2^{ii}	117.775(0)
I3^{iii}—Cu2—I3	112.575(0)	I2—Cu1—N2^{ii}	101.503(0)
I3—Cu2—N4	103.298(0)	I2^i—Cu1—N1	112.014(0)
Cu1—I1—Cu1^i	71.255(0)	I1^i—Cu1—N2^{ii}	109.258(0)

(i) -y, -x, 0.5-z; (ii) y, -1+x, 1-z; (iii) 1-y, 1-x, 0.5-z.

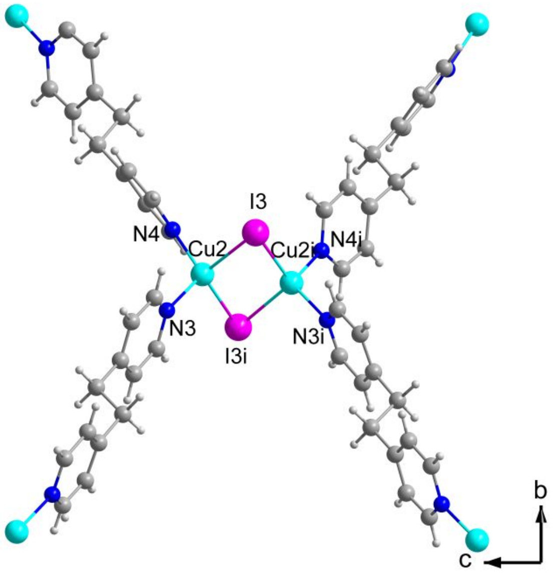

Figure S4. Second two-dimensional $[CuIbpe]_n$ network existing in **1** interpenetrated to that of Figure 1a with atom numbering scheme.

3.2. Photoluminescent studies

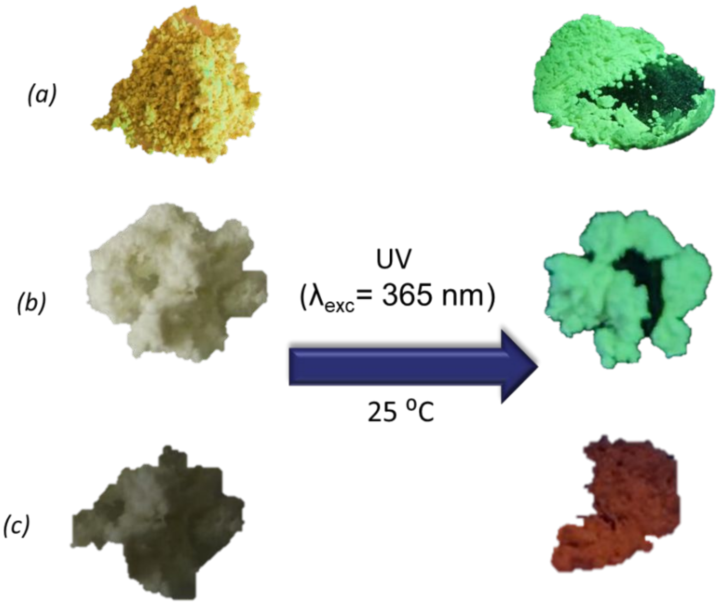

Figure S5. Green emission at λ_{exc} 365 nm of compounds **1** (a), **2** (b) and orange emission of **3** (c) at 25°C .

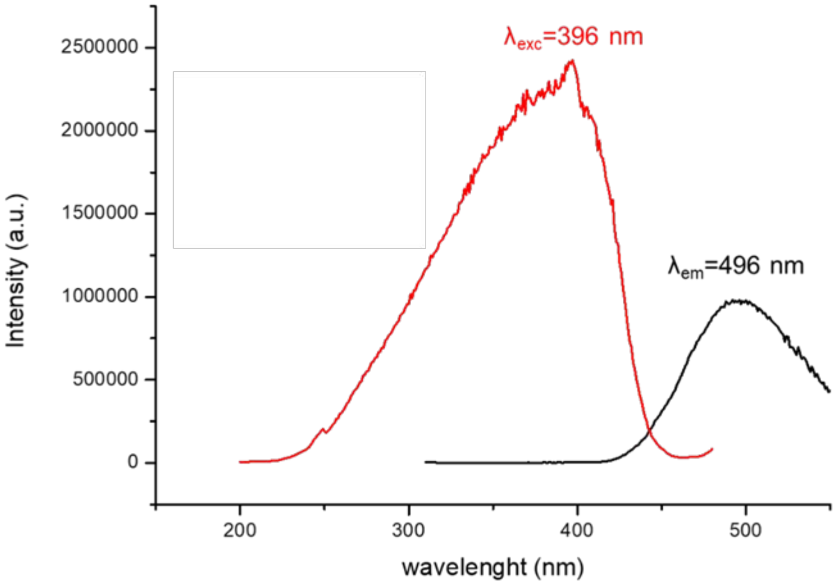

Figure S6. Emission (black) and excitation (red) spectra of the 2D CP **1.** (λ_{em}=496 nm, λ_{exc}=396 nm).

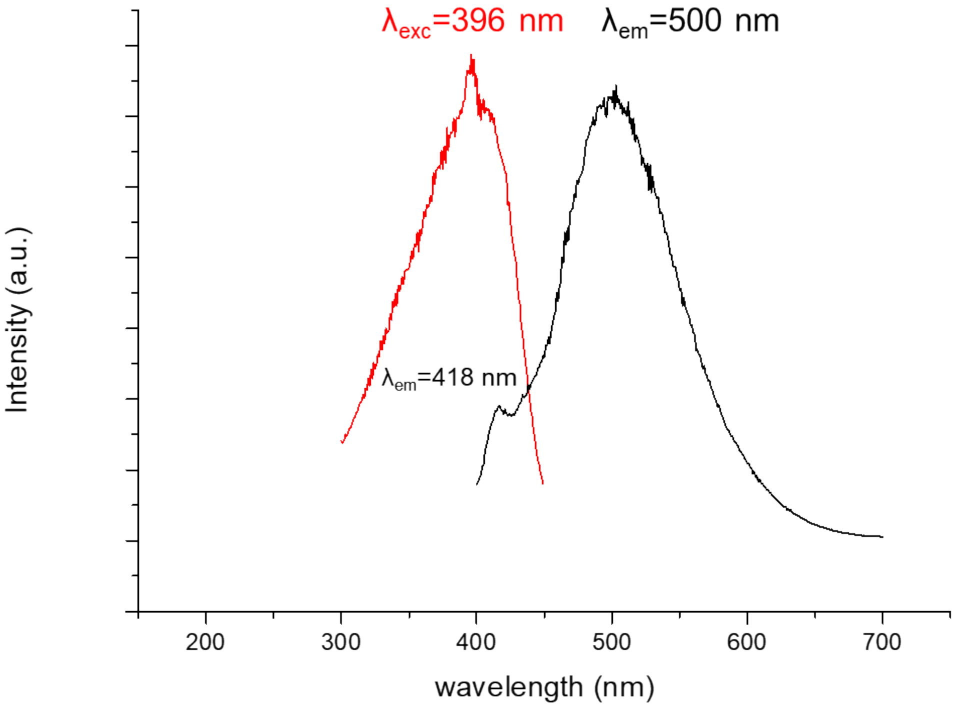

Figure S7. Emission (black) and excitation (red) spectra of the 2D CP **2.** (λ_{em}= 418 and 500 nm, λ_{exc}=396 nm).

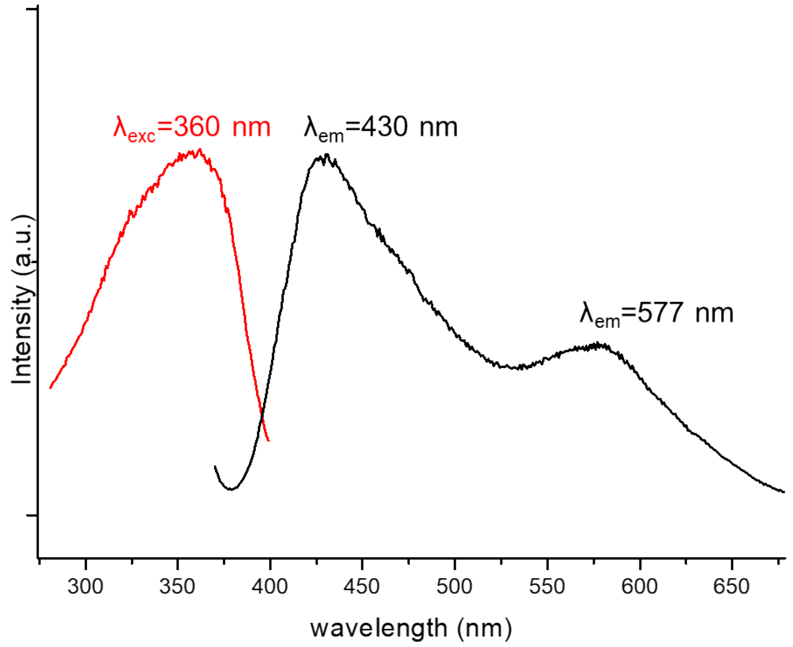

Figure S8. Emission (black) and excitation (red) spectra of the 2D **CP 3** (λ_{em}=430 nm and 577 nm, λ_{exc}=360 nm).

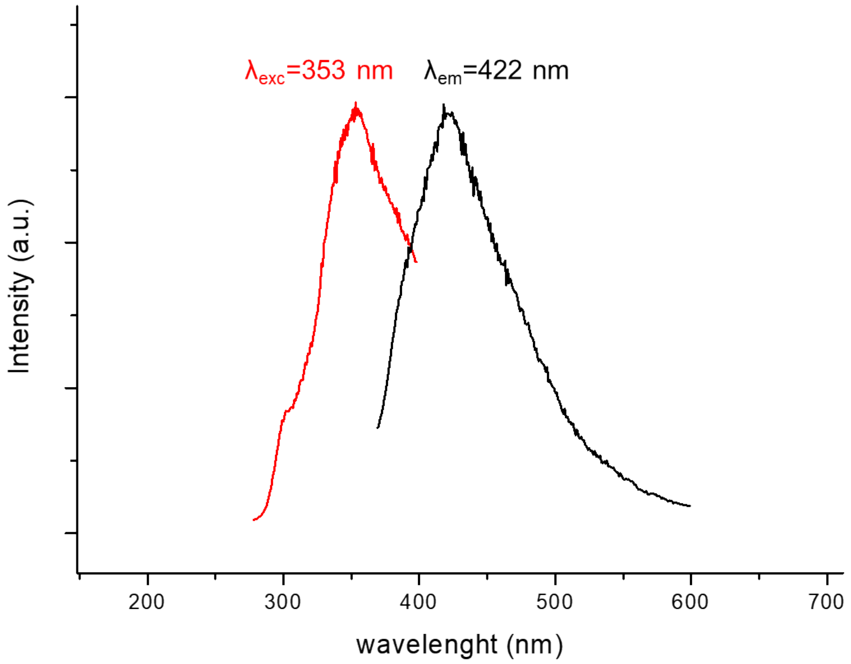

Figure S9. Emission (black) and excitation (red) spectra of the ligand 1,2-bis(4-pyridyl) ethane (bpe) (λ_{em}=422 nm, λ_{exc}=353 nm).

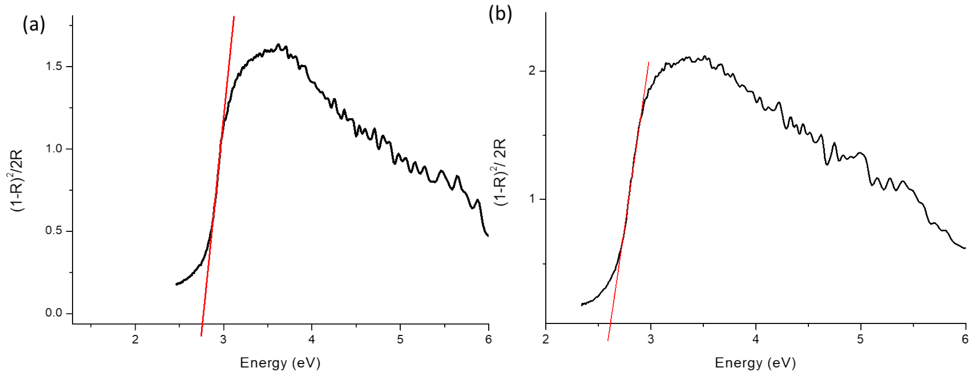

Figure S10. Optical absorption spectra of compounds **1** **(a)** and **2** (b) recorded at room temperature and converted to the Kubelka−Munk function. The band gaps were estimated from their absorption edges.

3.3 Thermal Phase transformation

Thermal stability studies

The thermo-gravimetric (TGA) of compound **1** shows 4 stages. The first mass loss of 11.7% occurs between 140 and 180 ºC. The second mass loss is equivalent to 13.3% and occurs between 180 ºC to 230 ºC. The sum of these mass losses is equivalent to half a ligand. The third mass loss of 24.5% occurs between 282 ºC and 400 ºC. This loss implies the loss of the bpe. The last mass loss of 44.3% occurs from 495 ºC to 680 ºC and corresponds to CuI (Figure S11a). The TGA of compound **2** presents 3 stages. The first mass loss of 19.2% occurs between 170 and 200 ºC. The second mass loss is equivalent to 26.3% and occurs between 200 ºC to 350 ºC. Probably indicative of the bpe consecutive loss. The last mass loss of 45.4% occurs from 440 ºC to 680 ºC and probably corresponds to CuI (Figure S11b).

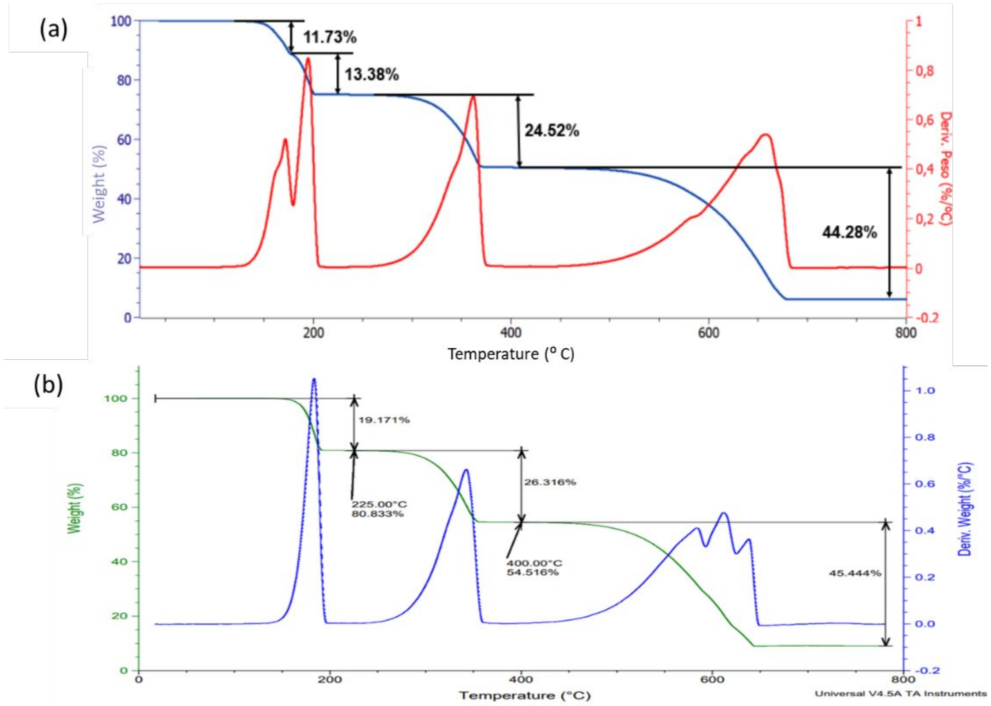

Figure S11. Thermogravimetric analysis of a) **1** (red and blue lines) and b) **2** (green and blue lines), represented in a temperature range from 20 ^{0}C to 800 ^{0}C, with a heating rate of 10 ^{0}C/min.

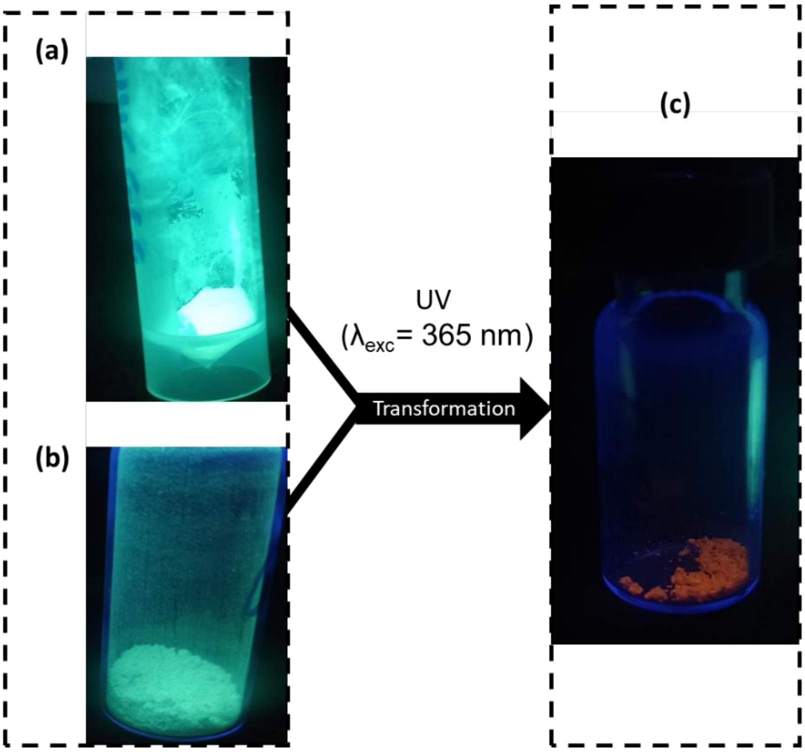

Figure S12. Under UV light: Green Emission of compounds **1** (a) and **2** (b) at 25ºC and orange emission of compounds **1** and **2** (c) after heating at 150 and 170^{0}C respectively.

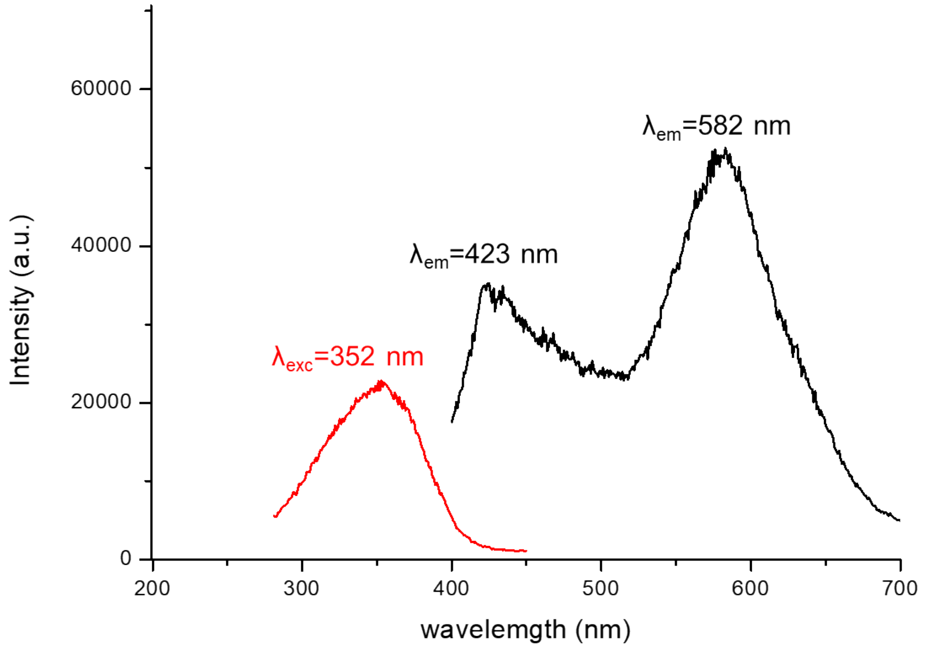

Figure S13. Emission (black) and excitation (red) spectra of the 2D CP **2.** λ_{em}=423 and 582 nm, λ_{exc}=352 nm. After heating 170 ^{0}C for 15 minutes.

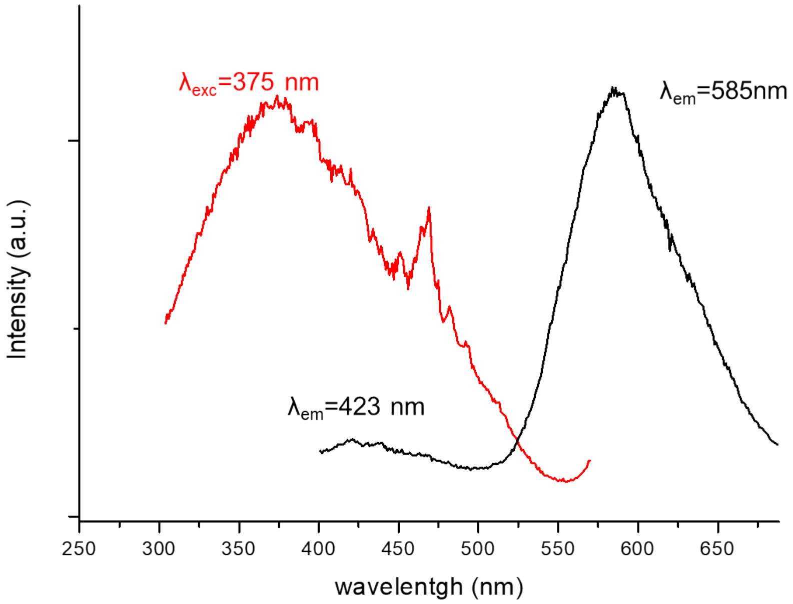

Figure S14. Emission (black) and excitation (red) spectra of the 2D CP **1**, λ_{em}=423 and 585 nm, λ_{exc}=375 nm. After heating 150 ^{0}C for 15 minutes.

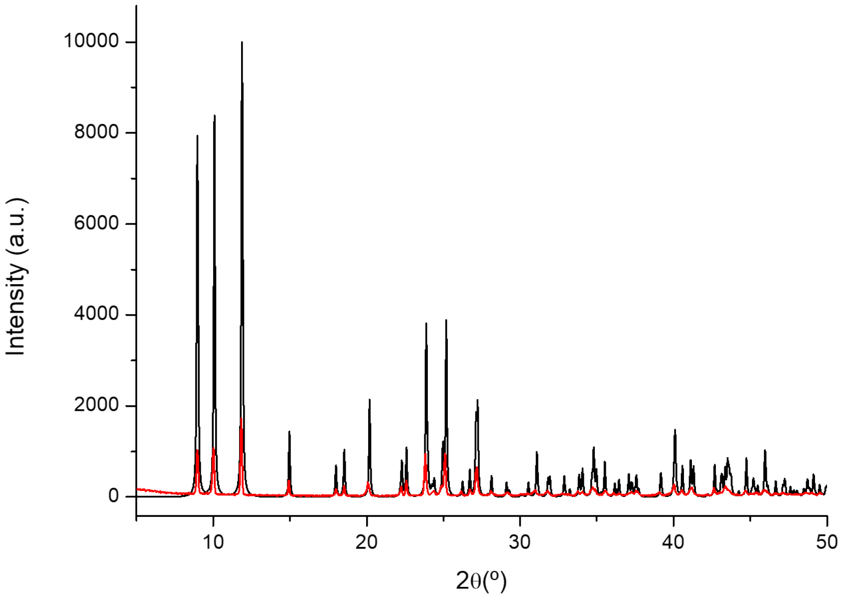

Figure S15. X-ray powder diffractogram of the compound **1** after heated at 150^{0}C (red line) compared with the X-ray powder diffractogram of compound **3** without heating (black line).

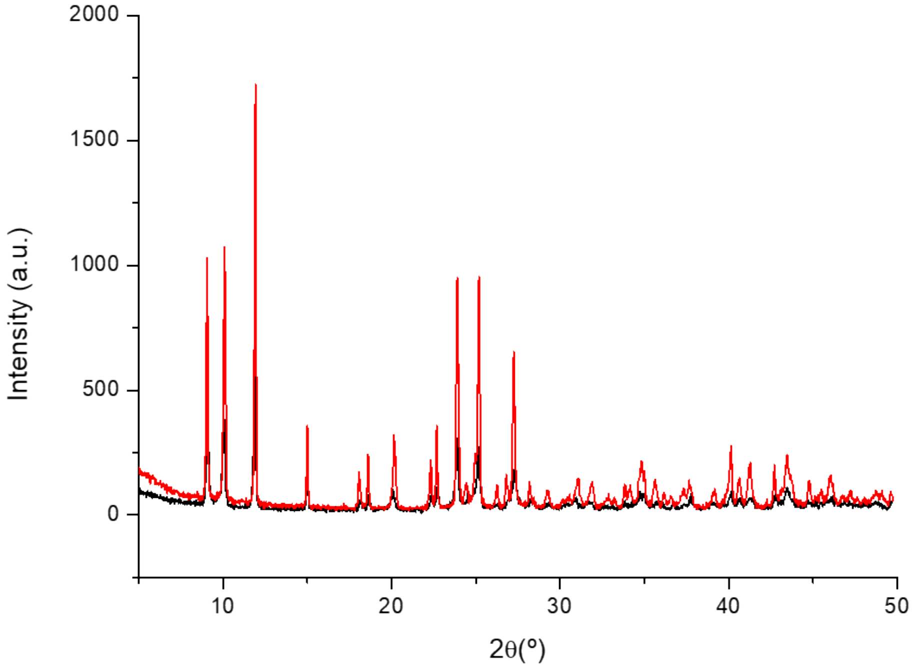

Figure S16. X-ray powder diffractogram of the compound **2** after heated at 170^{0}C (red line) compared with the X-ray powder diffractogram of compound **3** without heating (black line).

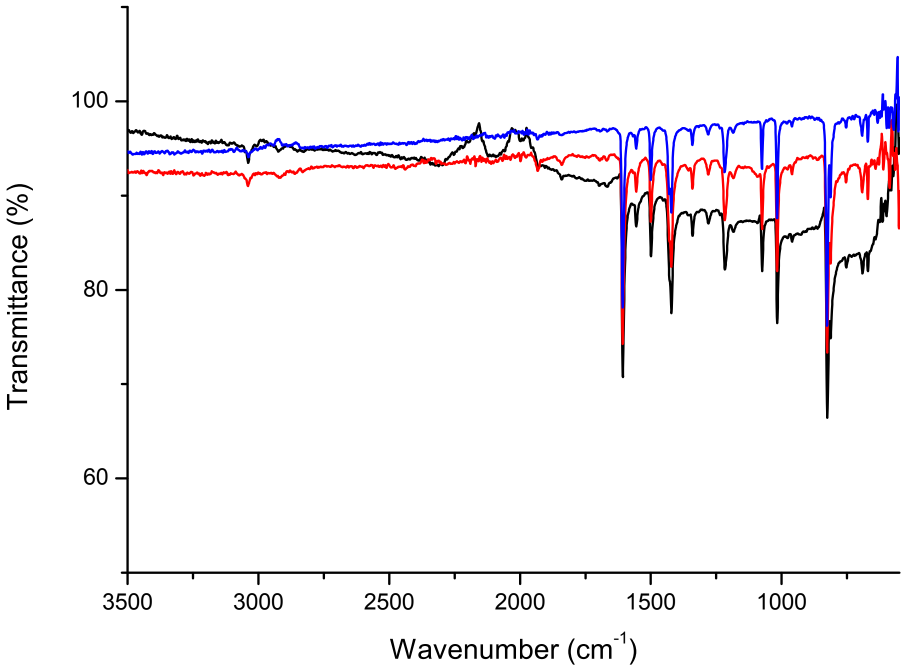

Figure S17. IR spectra of compounds **1** (blue line) and **2** (black line) after heating to 150 and 170^{0}C respectively comparing with compound **3** (red line).

3.4 Chemical Phase transformation

Dispersion studies

Table S3: Study of the stability of 10 mg of compounds **1**, **2**, and **3** in 2 mL of different organic solvents at 25^{0}C and 10 min at 700 rpm.

Solvents	**1**	**2**	**3**
Acetonitrile	Chemical Transformation	Chemical Transformation	Stable
Methanol	Partially stable	Stable	Stable
Dichloromethane	Chemical transformation	Chemical Transformation	Stable
Water	Stable	Stable	Stable

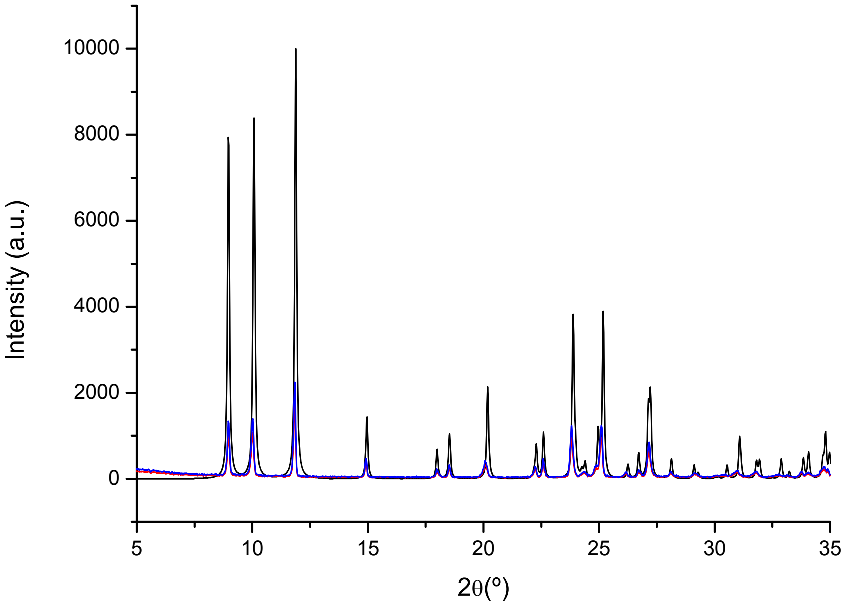

Figure S18. X-ray powder diffractogram at 25 ^{0}C after 30 minutes in acetonitrile or CH_2Cl_2 of compounds **1** (red) and **2** (blue) and imulated powder pattern of **3** (black).

Table S4. Study of the stability of compounds **1**, **2**, and **3** in different sonication conditions.

10 min sonication (40 kHz)	1 in H_2O/MeOH	2 in MeOH	3 in MeCN
40 %	Stable	Stable	Stable
60 %	Chemical transformation	Stable	Stable
80 %	Unstable	Stable	Stable

^{1}H-NMR study

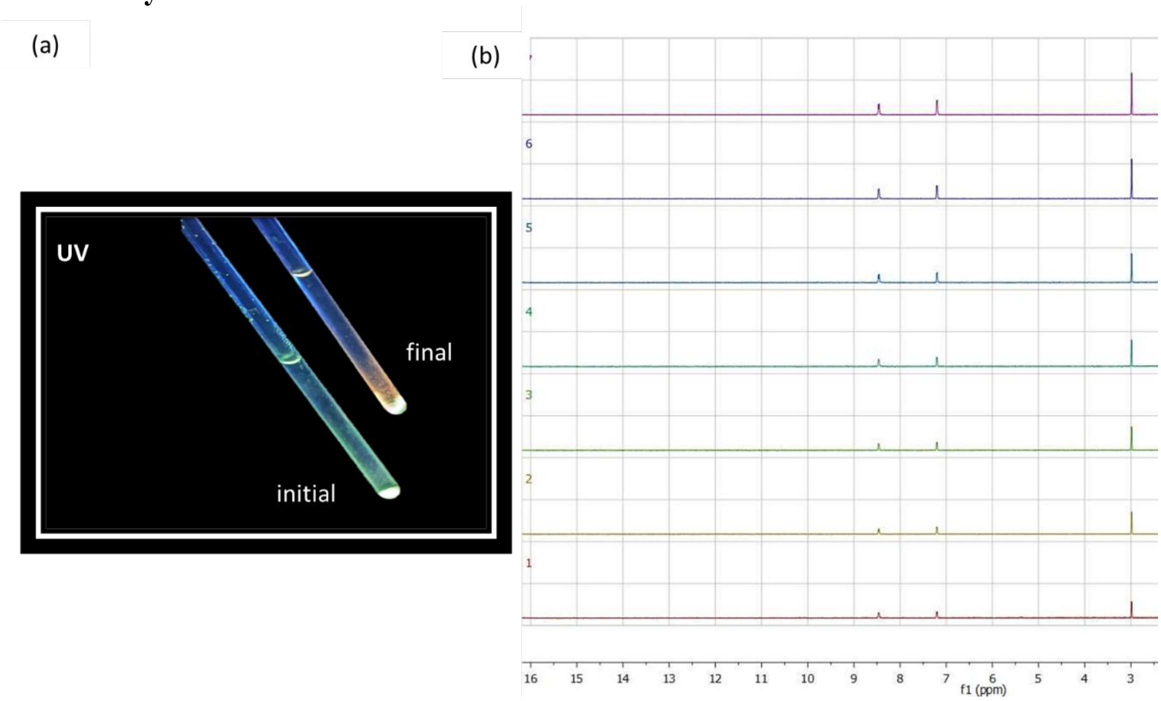

Figure S19. a) NMR tubes under UV light (λ=365 nm) and b) ^{1}H-NMR spectra of compounds **1** and **2** in CD_3CN recorded every 5 minute intervals from t= 0 min (1) to t= 35 min (7).

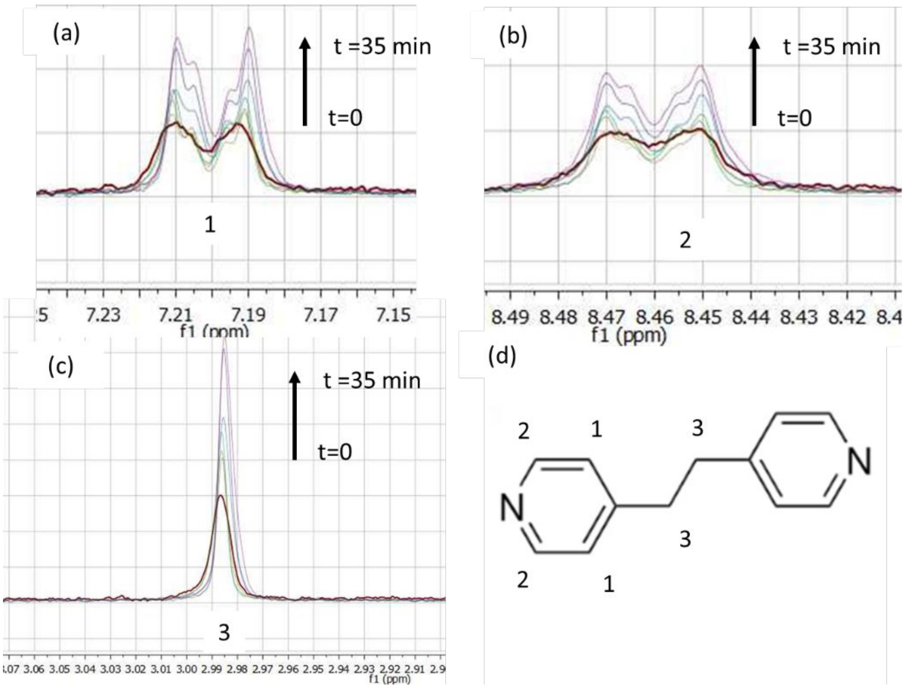

Figure S20. ^{1}H-NMR spectra of compound **1** and **2** in CD_3CN recorded every 5 minutes from time 0 to time 35 minutes: a) doublet corresponding to protons marked as 1 in the ligand (*bpe*), b) doublet corresponding to protons marked as 2 in the ligand (*bpe*), c) singlet corresponding to protons marked as 3 in the ligand (*bpe*) and d) *bpe* ligand.

Morphological study by SEM

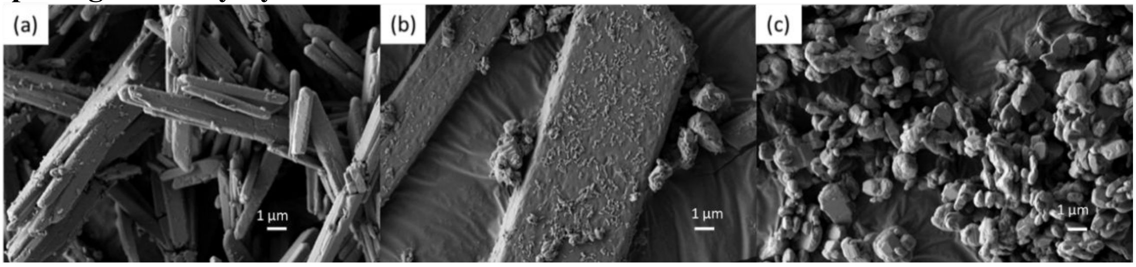

Figure S21. SEM images 5.0K X of depositions (1%): **1** (a), **2** (b) and **3** (c).

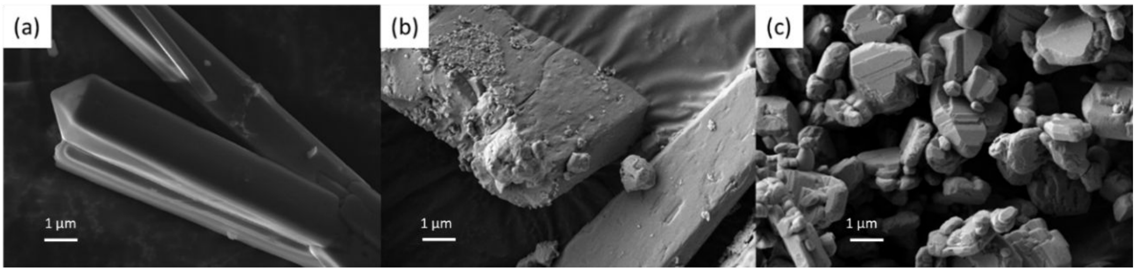

Figure S22. SEM images 10.0K X of depositions (0.5%): **1** (a), **2** (b) and **3** (c).

3.6. EQE Studies and Transmittance measurements.

Table S5: Area values of the integral of the curve obtained in the EQE of compounds 1-3 and the minimodule (m-m) after encapsulation.

m-m (a.u.)	1@0.5% (a.u.)	1@1% (a.u.)	2@0.5% (a.u.)	2@1% (a.u.)	3@0.5% (a.u.)	3@1% (a.u.)
526.6861	519.9847	460.7093	520.2270	457.2371	490.5563	484.5797

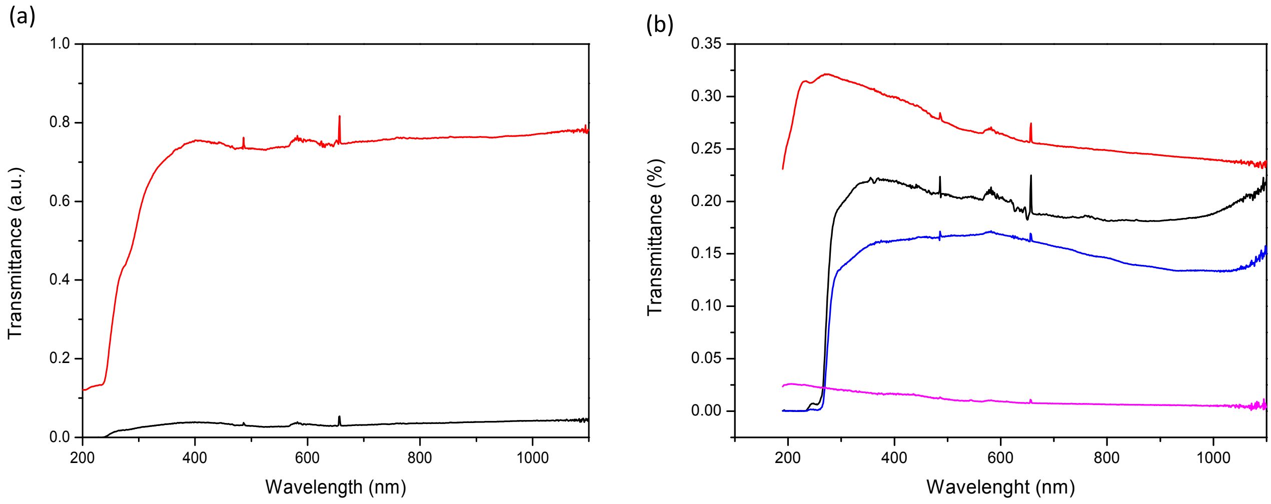

Figure S23. Transmittance of a) EVA before (black) and after encapsulation (red) and b) compound 2@0.5% before (black) and after (red) and 2@1% before (pink) and after (blue) encapsulation process.

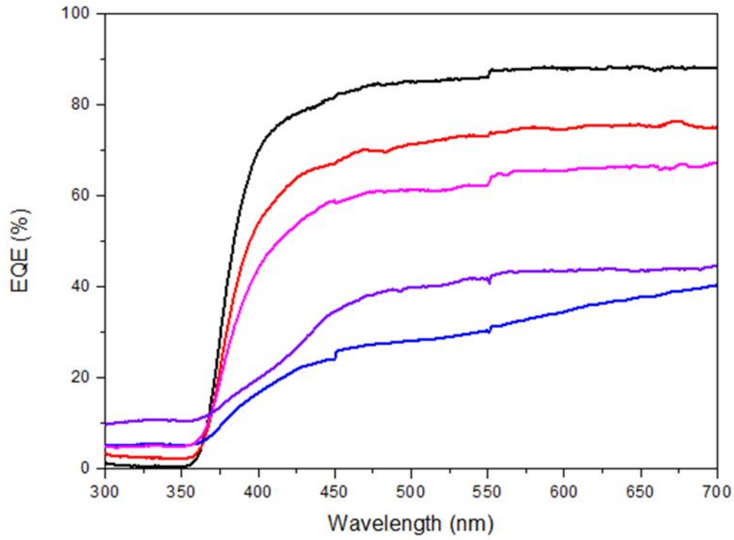

Figure S24. EQE before encapsulation of mini-module (black) and compounds: **1**@0.5% (red) and **1**@1% (blue); **2**@0.5 (pink) and **2**@1% (violet)..

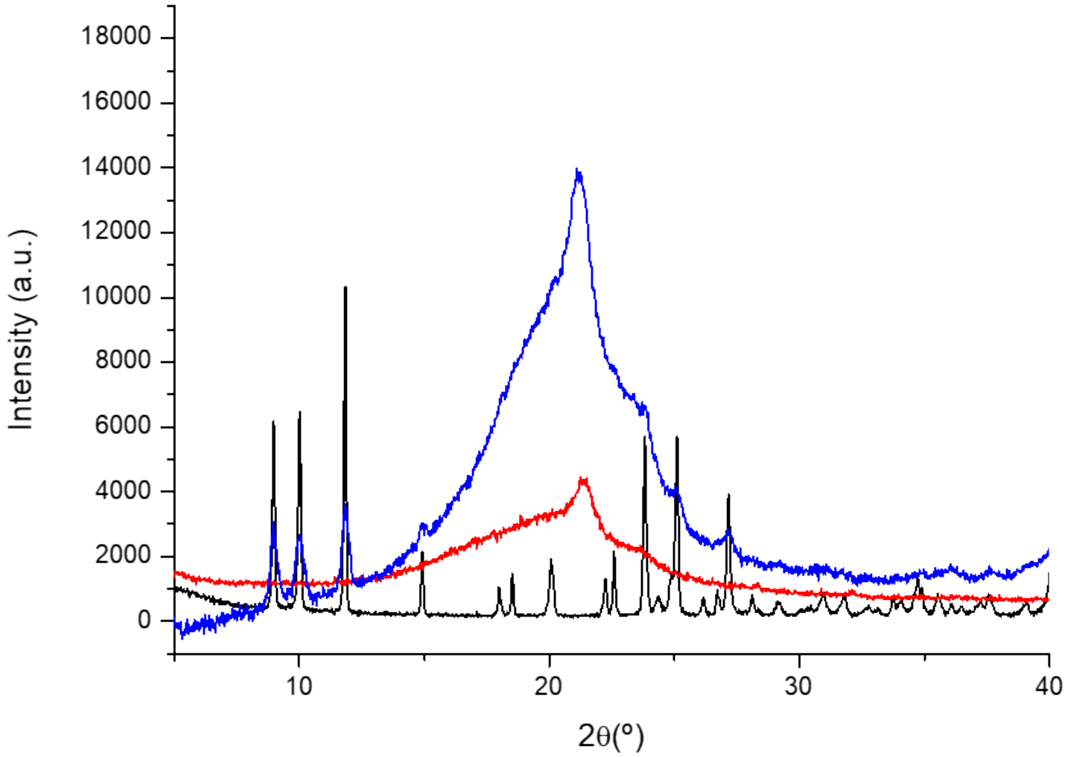

Figure S25. X-ray powder diffractogram of 1@1% (blue) 2 moths later after the encapsulation process at 150 degrees, compound 3 (black) and EVA (red).

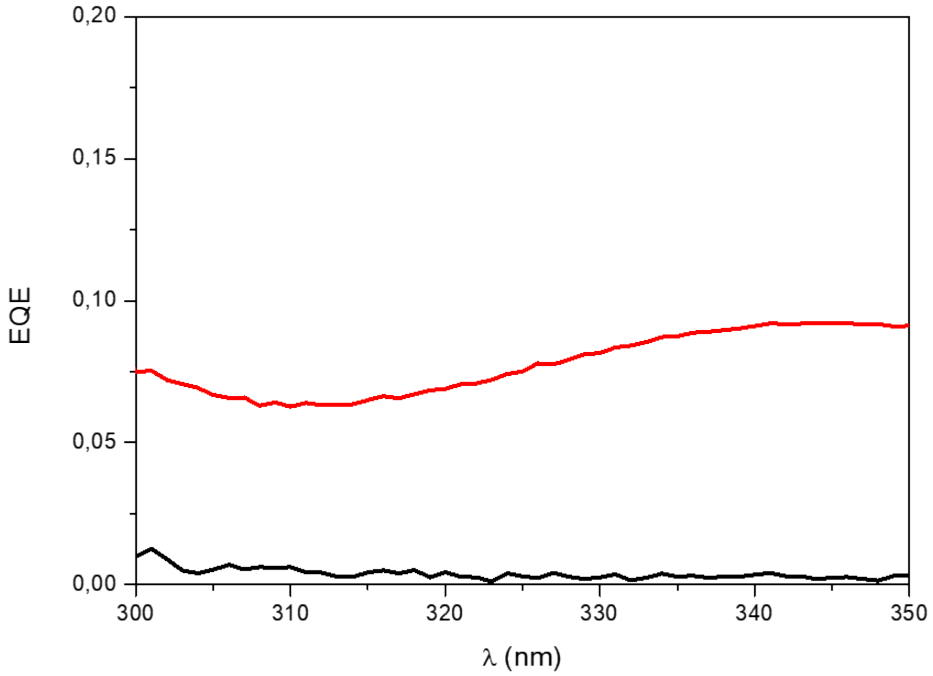

Figure S26. EQE of compound [Eu(tta)$_3$(phen)]@EVA-5% (red green) in UV region after encapsulation versus mini-module (black).
